# Molecularly Engineered Alicyclic Organic Spacers for 2D/3D Hybrid Tin‐based Perovskite Solar Cells

**DOI:** 10.1002/smll.202405598

**Published:** 2024-09-03

**Authors:** Jinhyeok Choi, Jimin Kim, Minyoung Jeong, Byeongchan Park, Seunghyun Kim, Jisang Park, Kilwon Cho

**Affiliations:** ^1^ Department of Chemical Engineering Pohang University of Science and Technology Pohang 37673 South Korea

**Keywords:** 2D/3D heterostructures, defect passivation, hydrogen bonding, rigidity, tin‐based perovskite solar cells

## Abstract

The high defect density and inferior crystallinity remain great hurdles for developing highly efficient and stable Sn‐based perovskite solar cells (PSCs). 2D/3D heterostructures show strong potential to overcome these bottlenecks; however, a limited diversity of organic spacers has hindered further improvement. Herein, a novel alicyclic organic spacer, morpholinium iodide (MPI), is reported for developing structurally stabilized 2D/3D perovskite. Introducing a secondary ammonium and ether group to alicyclic spacers in 2D perovskite enhances its rigidity, which leads to increased hydrogen bonding and intermolecular interaction within 2D perovskite. These strengthened interactions facilitate the formation of highly oriented 2D/3D perovskite with low structural disorder, which leads to effective passivation of Sn and I defects. Consequently, the MP‐based PSCs achieved a power conversion efficiency (PCE) of 12.04% with superior operational and oxidative stability. This work presents new insight into the design of organic spacers for highly efficient and stable Sn‐based PSCs.

## Graphical abstract

The introduction of morpholinium iodide (MPI) as an alicyclic organic spacer in 2D/3D perovskite solar cells enhances hydrogen bonding, and intermolecular interactions within 2D perovskite structure. This reduces structural disorder and effectively passivates defects, achieving a power conversion efficiency of 12.04% with improved stability. This study offers new insights into designing organic spacers for Sn‐based PSCs.

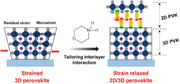

## 1 Introduction

Metal halide perovskites have attracted significant attention as promising materials for next‐generation photovoltaics because of their superior optoelectronic properties.^[^ ^1^, ^2^, ^3^, ^4^, ^5^, ^6^, ^7^, ^8^ ^]^ Notably, lead (Pb)‐based perovskite solar cells (PSCs) have shown remarkable progress, achieving a power conversion efficiency (PCE) of 26.1%.^[^ ^9^ ^]^ However, the toxicity and the environmental hazard of Pb remain a major obstacle to the commercialization of PSCs.^[^ ^10^, ^11^ ^]^ Among the various candidates for replacing Pb with other metal elements, tin (Sn)‐based perovskites have strong potential for promising candidates because of their small exciton binding energy, high carrier mobility, and narrow bandgap of 1.4 eV, which is an optimal bandgap for approaching the Shockley–Queisser limit.^[^ ^12^, ^13^, ^14^ ^]^ However, the efficiency and stability of Sn‐based PSCs still lag behind that of Pb‐based PSCs, which results from the high defect density, the facile oxidation of Sn^2+^, and poor film quality with low crystallinity due to uncontrollable crystallization.^[^ ^15^, ^16^, ^17^, ^18^, ^19^ ^]^

To address these issues, considerable efforts have been devoted to the development of highly efficient and stable Sn‐based PSCs.^[^ ^20^, ^21^, ^22^, ^23^ ^]^ In particular, incorporating bulky ammonium cations (*e.g*., butylammonium (BA),^[^ ^24^ ^]^ anilinium (AN),^[^ ^25^ ^]^ phenylethylammonium (PEA),^[^ ^26^, ^27^ ^]^ and 4‐fluoro‐phenethylammonium (FPEA))^[^ ^28^ ^]^ as 2D Ruddlesden–Popper spacers to form 2D/ 3D hybrid perovskite has shown great advantages in protecting the 3D Sn‐based perovskite from moisture, suppressing the oxidation of Sn^2+^, and passivating structural defects. However, there has been limited discussion about the organic spacers in Sn‐based perovskite; the variety of organic spacers has mostly been confined to aliphatic and aromatic types. In contrast, studies on a wide range of 2D spacers have been extensively explored in Pb‐based perovskites. Among the various candidates for 2D spacers, alicyclic spacers show great potential because of their moderate rigidity. The stability of 2D perovskites is influenced by the rigidity of the organic spacers; alicyclic spacers, with their medium rigidity, demonstrate greater structural stability compared to the high rigidity of aromatic spacers and the low rigidity of aliphatic spacers.^[^ ^29^ ^]^ Despite the great potential of the alicyclic spacers, further studies are still required to effectively apply alicyclic spacers to Sn‐based perovskites. In Sn‐based perovskite, the 2D layer of cyclohexylammonium (CHA) in 2D/3D perovskite still exhibited a randomly oriented crystal with poor structural properties,^[^ ^25^ ^]^ modulating the properties of CHA is essential to enable the use of alicyclic spacers in Sn‐based perovskites. However, there has been limited discussion regarding alicyclic ammonium spacers in Sn‐based perovskite when compared to their alkyl or aromatic ammonium spacers.

Here, for the first time, we report a novel alicyclic organic spacer, morpholinium (MP), for developing structurally stabilized 2D/3D Sn‐based perovskites. We compared three alicyclic organic spacers (i.e., CHA, piperidinium (PD), and MP) to determine how the secondary ammonium group and inclusion of a heteroatom in spacer cations affect the structural properties and defect passivation of 2D Sn‐based perovskites. Introducing a secondary ammonium and ether group in alicyclic spacers exploited the rigidity of spacers and thereby increased the structural rigidity of 2D perovskite. This led to increased robustness of hydrogen bonding between the ammonium group and the inorganic framework of the perovskite structure. In addition, the O atoms in the organic spacers form weak hydrogen bonds with C–H in adjacent organic spacers, enhancing the intermolecular interaction between organic spacers. These combined effects led to the formation of highly oriented 2D/3D perovskite crystals with decreased structural disorder. Moreover, MP‐based 2D/3D perovskite significantly passivated structural defects, and thereby effectively mitigated nonradiative recombination and facilitated interfacial charge extraction. Consequently, the MP‐based 2D/3D PSCs exhibited a best‐performing PCE of 12.04% with superior oxidative and operational stability. The unencapsulated devices retained 77% of their initial PCE after 250 h exposure to air (20–25 °C, 35–40 RH%), and 71% of their initial PCE after 500 h under continuous light illumination at the maximum power point (MPP) under a N_2_ atmosphere.

## 2 Results and Discussion

**Figure 1:**
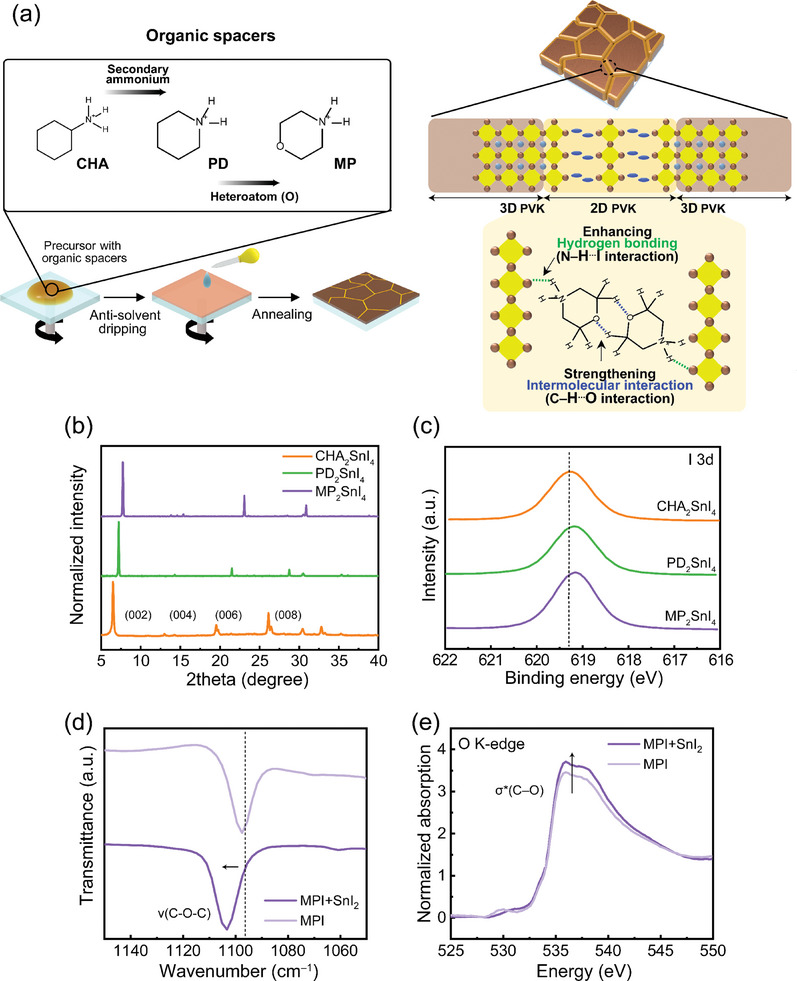
a) Left: Deposition process of 2D/3D perovskite using different organic spacers; cyclohexylammonium (CHA), piperidinium (PD), and morpholinium (MP). Right: Schematic illustration of the crystal structure of MP‐based 2D/3D perovskite. b) XRD patterns of 2D perovskite films. c) XPS I 3*d* peak of pure 2D perovskite films. d) FT‐IR and e) XANES of pure MPI and the mixture of MPI and SnI_2_.

2D/3D perovskites were prepared by mixing 95 mol% of 3D formamidinium tin triiodide (FASnI_3_) precursor with 5 mol% of 2D perovskite precursor (CHA_2_SnI_4_, PD_2_SnI_4_, or MP_2_SnI_4_). A one‐step antisolvent dripping method was used to fabricate the 2D/3D perovskite film, followed by a thermal annealing process. A schematic of the investigated MP‐based 2D perovskite is depicted (**Figure** **1**a). The X‐ray diffraction (XRD) patterns of 2D perovskite films revealed characteristic peaks of the 2D perovskite structures, confirming the influence of organic spacers in forming 2D perovskites (Figure 1b). The *d*‐spacings of the 2D perovskites were calculated to be 13.57, 12.21, and 11.39 Å for CHA‐, PD‐ and MP‐based 2D perovskite films, respectively. The lower *d*‐spacings of MP‐based 2D perovskite show its more dense structure. To determine the influence of *d*‐spacings of 2D perovskite on the interaction between organic spacers and the perovskite, the 2D perovskite films were characterized by X‐ray photoelectron spectroscopy (XPS) (Figure 1c). The I 3*d* peaks in the spectra of PD‐based and MP‐based 2D perovskite films were positioned at lower binding energies than those in the spectrum of the CHA‐based 2D perovskite films. The decreased binding energy of the I 3*d* peak indicates an increase in the bond length of Sn–I,^[^ ^30^ ^]^ which is attributed to the stronger interaction between secondary ammonium organic spacers and [SnI_6_]^4–^ octahedra than primary ones.^[^ ^31^, ^32^ ^]^ This result could be attributed to the reduced *d*‐spacing in 2D perovskites, leading to a closer proximity between the organic spacers and the [SnI_6_]^4–^ octahedra, consequently enhancing the hydrogen bonding between these components. This could be further verified by the Fourier transform infrared (FT‐IR) spectra, which show a large N–H bending vibrational peak shift to a lower wavenumber upon the addition of SnI_2_ into PDI and MPI than CHAI (Figure S1, Supporting Information).

The study of the crystal structure of MPI revealed that MP^+^ cations in MPI are connected through weak C–H–O hydrogen bonds.^[^ ^33^, ^34^ ^]^ In forming MP‐based 2D perovskites, these C–H–O interactions are retained within the 2D framework. Conversely, in the absence of 2D perovskite formation, alternative interactions such as N–H–O or Sn^2+^–O become prominent. FT‐IR spectra of pure MPI and a mixture of MPI and SnI_2_ indicated that the addition of SnI_2_ to MPI maintains the C–H–O interactions, as evidenced by the shift in the C–O–C stretching vibration to a higher wavenumber (Figure 1d). This suggests that a mixture of MPI and SnI_2_, the dominant interaction is still C–H–O, although weaker than in pure MPI crystals due to less dense packing in the 2D perovskite structure. Further investigation using X‐ray absorption near‐edge structure (XANES) spectra on the same films showed a peak at ≈536 eV in the oxygen K‐edge XANES spectra, related to antibonding C─O bonds (σ^*^(C–O)) in both pure MPI and the mixture of MPI and SnI_2_ (Figure 1e).^[^ ^35^ ^]^ The higher intensity of this peak in the mixture indicates a change in charge distribution around the C─O bonds of MP^+^ cations when SnI_2_ is added.^[^ ^36^ ^]^ This result suggests that the formation of 2D perovskites weakens the C–H–O interaction between adjacent MP^+^ ions, which may lead to stronger C─O bonds. These results confirm that the robust molecular interaction was facilitated by the C–H–O intermolecular interaction between the spacer.

**Figure 2:**
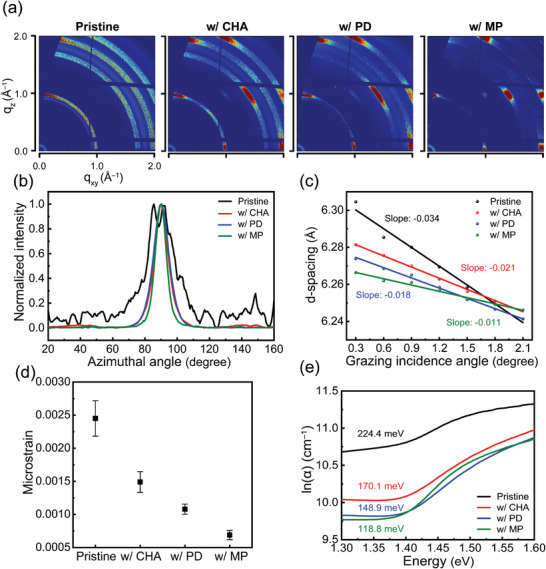
a) GIWAXS (incidence angle 0.3°) of 3D and 2D/3D perovskite films. b) Polar profiles of the azimuthal angle with 3D and 2D/3D perovskite films. c) *d*‐spacing value of the (100) plane as a function of grazing incidence angle for the 3D and 2D/3D perovskite films obtained from GIXRD spectra. d) Lattice microstrain of 3D and 2D/3D perovskite films obtained from Williamson–Hall plot. e) Urbach energy of 3D and 2D/3D perovskite films extracted from absorption spectra.

The XRD patterns of 2D/3D perovskite structures with 20 mol% of organic spacers shows that specific low‐angle peaks (2*θ* < 10°), which provide evidence of the presence of 2D/3D heterostructures (Figure S2a, Supporting Information). Further analysis using time‐of‐flight secondary ion mass spectrometry (TOF‐SIMS) showed that the 2D perovskite phase in these films is predominantly located at the surface and grain boundaries (Figure S3, Supporting Information). However, in the scanning electron microscopy (SEM) images of 2D/3D perovskite films with 5 mol% organic spacers, no apparent heterogeneous phase is observed on the surface of the 2D/3D perovskite films. This could be attributed to the fact that the 2D/3D perovskite film consists of a very thin layer of 2D perovskite at the surface and grain boundaries of the films (Figures S4 and S5, Supporting Information).^[^ ^37^ ^]^ Moreover, the grain size of the 2D/3D perovskite slightly decreases compared to that of the 3D perovskite. We speculate that the organic spacers interact with Sn^2+^, potentially retarding the reaction of FASnI_3_ and SnI_2_, which leads to the reduced grain size of the 2D/3D perovskite.^[^ ^38^, ^39^ ^]^ Meanwhile, the 2D/3D perovskite films with 5 mol% of organic spacers displayed more intense (*h*00) peaks than the 3D pristine films (Figure S2b, Supporting Information), suggesting a preferred out‐of‐plane growth orientation in the 2D/3D heterostructures. To further probe the oriented growth of 2D/3D perovskite films, grazing incidence wide‐angle X‐ray scattering (GIWAXS) was performed. The GIWAXS patterns showed that the 3D pristine film had random crystal orientation, while the 2D/3D perovskite films displayed sharp, distinct Bragg spots, particularly in the MP‐based films which were clearer than others (**Figure** **2**a). In comparison, CHA‐based films showed peaks at specific angles ≈of 145°, whereas PD‐ and MP‐based films had a single peak, indicating higher crystallinity in MP‐based films (Figure 2b). Consequently, the strong interlayer interaction within a 2D perovskite structure induces the oriented growth of the perovskite and tightens the overall 2D/3D heterojunction structure, thereby promoting the formation of continuous charge transport pathways and enabling efficient charge extraction.^[^ ^40^ ^]^

The residual strain, which inevitably arises because of the difference in the thermal expansion coefficients of the perovskite and the substrates, induces the formation of defects and lattice distortion and thereby deteriorates the efficiency and stability of PSCs.^[^ ^41^, ^42^ ^]^ To gain insight into the effect of different organic spacers on the distribution of interfacial strain in perovskite, we performed grazing‐incidence X‐ray diffraction (GIXRD) measurements of 3D pristine and 2D/3D perovskite films (Figure 2c; Figure S6, Supporting Information). A shift in the (100) peaks to higher angles with increased incident angles was observed, which indicates a decrease in interplanar spacing and the presence of compressive strain in these films. Notably, the MP‐based 2D/3D perovskite film showed a smaller peak shift, suggesting less interfacial strain compared to others. We also quantified the microstrain (*ε*) of the 3D pristine film and 2D/3D perovskite films (Figure 2d), which was quantified using Williamson–Hall plots (Figure S7, Supporting Information)

(1) $$\beta \;\cos\theta =\;\frac{K\lambda }{L}+4\varepsilon \sin\theta$$

where *θ* is the Bragg angle, *β* is the line broadening measured for each (*hkl*) plane at the FWHM in radians, which is calibrated by the instrumental line broadening, *K* is a shape factor, *λ* is the X‐ray wavelength, and *L* is the mean size of the crystallites. Microstrain arises from variations in the lattice spacing, which results from crystal imperfection and structural disorders, including vacancies and microdomains.^[^ ^43^, ^44^ ^]^ The calculated *ε* values were 2.45 × 10^−3^ for the 3D pristine, and 1.49 × 10^−3^, 1.08 × 10^−3^, and 6.89 × 10^−4^ for the CHA‐based, PD‐based, and MP‐based 2D/3D perovskite films, respectively. The MP‐based 2D/3D perovskite film exhibited substantial relaxation of microstrain, followed by the PD‐based, CHA‐based 2D/3D perovskite, and the 3D pristine perovskite. Meanwhile, the structural disorder in perovskite can create disorder near the band edge and thereby cause nonradiative recombination and the loss of open‐circuit voltage (*V*_oc_).^[^ ^45^, ^46^ ^]^ To quantify the energetic disorder in these films, we calculated the Urbach energy (*E*_u_) (Figure 2e), which can be extracted from UV–vis spectra (Figure S8a, Supporting Information), which follows the equation

(2) $$A\;=A_{0}\;exp\left(\frac{E}{E_{u}}\right)$$

where *A* is the absorbance, *A*_0_ is a constant, and *E* is the energy of a photon. The extracted *E*_u_ values were 224.4 meV for the 3D pristine film and 170.1, 148.9, and 118.8 meV for the CHA‐based, PD‐based, and MP‐based 2D/3D perovskite films, respectively, revealing a decreasing trend of energetic disorder.^[^ ^47^, ^48^, ^49^ ^]^ This result agrees well with the observed trend in the reduction of microstrain. These results indicate that the synergetic effect of enhanced hydrogen bonding and C–H–O intermolecular interaction in the 2D perovskite phase mitigates both structural and energetic disorder in the 2D/3D perovskite films.

**Figure 3:**
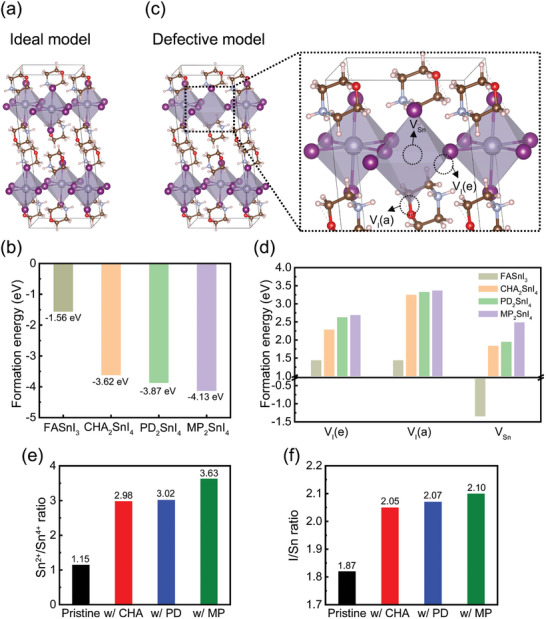
a) Structural model of MP‐based 2D perovskite. b) Calculated formation energy of 3D and pure 2D perovskite. c) Structural model of 2D MP‐based perovskite with vacancies. V_I_(e): neutral I vacancies in the equatorial position; V_I_(a): neutral I vacancies in the apical position; V_Sn_: neutral Sn vacancies; d) Calculated vacancy formation energy of 3D and pure 2D perovskite. e) Sn^2+^/Sn^4+^ ratio obtained from deconvoluted XPS Sn 3*d* peaks of 3D and 2D/3D perovskite films. f) I/Sn ratio obtained from XPS Sn 3*d* and I 3*d* peaks of 3D and 2D/3D perovskite films.

To understand how different organic spacers in 2D perovskites affect the structural stability of perovskite structures, density functional theory (DFT) simulations were conducted on 3D FASnI_3_ perovskite and CHA‐based, PD‐based, and MP‐based 2D perovskites (**Figure** **3**a; Figure S9a, Supporting Information). After full geometric relaxation of these perovskite structures, we selected the most stable structure and then calculated the formation energy (*E*_f_) for each structure (Figure 3b). The negative *E*_f_ values indicated energetically favorable formation for all structures,^[^ ^50^ ^]^ with the absolute value of *E*_f_ being higher for MP‐based 2D perovskites, suggesting their superior stability. Meanwhile, the shortest distance between the O atom of one MP^+^ and the H atom of the C–H of another MP^+^ is 2.88 Å (Figure S9b, Supporting Information), which matches the range expected for weak hydrogen bonding,^[^ ^51^ ^]^ therefore, this result suggests that weak hydrogen bonding will occur between neighboring MP^+^ moieties. By contrast, the shortest distance between C–H–O in MPI is 2.52 Å,^[^ ^33^ ^]^ which suggests that the intermolecular interaction between two MP^+^s in the MP‐based 2D perovskite is weaker than that in MPI. This result is consistent with the peak shift observed in the FT‐IR spectrum and the intensity change in the XANES spectrum of the MP‐based 2D perovskite film. Hydrogen bonding and intermolecular interaction play an important role in stabilizing of the 2D perovskite structure,^[^ ^52^ ^]^ which implies that the strengthened intermolecular interaction and hydrogen bonding efficiently stabilize the MP‐based 2D perovskite structure.

In addition, the formation of structural defects was investigated by constructing defective models with neutral Sn vacancies (V_Sn_) and neutral I vacancies at both apical (V_I_ (a)) and equatorial positions (V_I_ (e)) (Figure 3c,d; Table S1, Supporting Information). Calculations showed that V_Sn_ are more likely in 3D perovskites than in 2D,^[^ ^53^ ^]^ particularly in MP‐based 2D perovskites. Also, the *E*_f_ values for V_I_ were higher in 2D perovskites than in 3D, with the order of CHA‐based < PD‐based < MP‐based 2D perovskite. This trend indicates a decrease in structural defects in MP‐based 2D perovskite and is consistent with the reduced microstrain in the MP‐based 2D/3D perovskites. On the basis of these results, we posit that the thermodynamic instability and the formation of undesirable traps in the Sn‐based perovskite films can be reduced by incorporating structurally stabilized 2D perovskites into 3D perovskites.

To verify that the formation of structural defects in the 2D/3D perovskite films was suppressed, we conducted XPS measurements of films of the 3D perovskite and the 2D/3D perovskites with different organic spacers. We deconvoluted the Sn 3*d* XPS spectra into those of Sn^2+^ and Sn^4+^ species to compare the Sn^2+^/Sn^4+^ ratio in each film (Figure S10a, Supporting Information). The ratios were 1.15 in 3D pristine perovskite film and 2.98, 3.02, and 3.63 in the CHA‐based, PD‐based, and MP‐based 2D/3D perovskite films, respectively (Figure 3e). The suppressed oxidation of Sn^2+^ is conspicuous in the MP‐based 2D/3D perovskite film and is consistent with the high *E*_f_ of V_Sn_ in the MP‐based 2D perovskite. We also examined the I‐to‐Sn (I/Sn) atomic ratio in each film to evaluate the stoichiometric compositions of the surface of each film (Figure 3f; Figure S10b, Supporting Information). The ideal stoichiometric I/Sn ratio should be close to 3, however, it was 1.87 in the 3D pristine film and 2.05, 2.07, and 2.10 in the CHA‐based, PD‐based, and MP‐based 2D/3D perovskite films, respectively. The nonstoichiometric composition of the surface of the perovskite is largely attributed to halide deficiency resulting from the halide vacancies.^[^ ^54^ ^]^ The higher I/Sn ratio in the MP‐based 2D/3D perovskite film compared with that in the 3D pristine indicates that the MP‐based 2D/3D perovskite had fewer I vacancies at the surface than the 3D pristine, which is also consistent with the high *E*_f_ of V_I_ in 2D/3D perovskite film.

**Figure 4:**
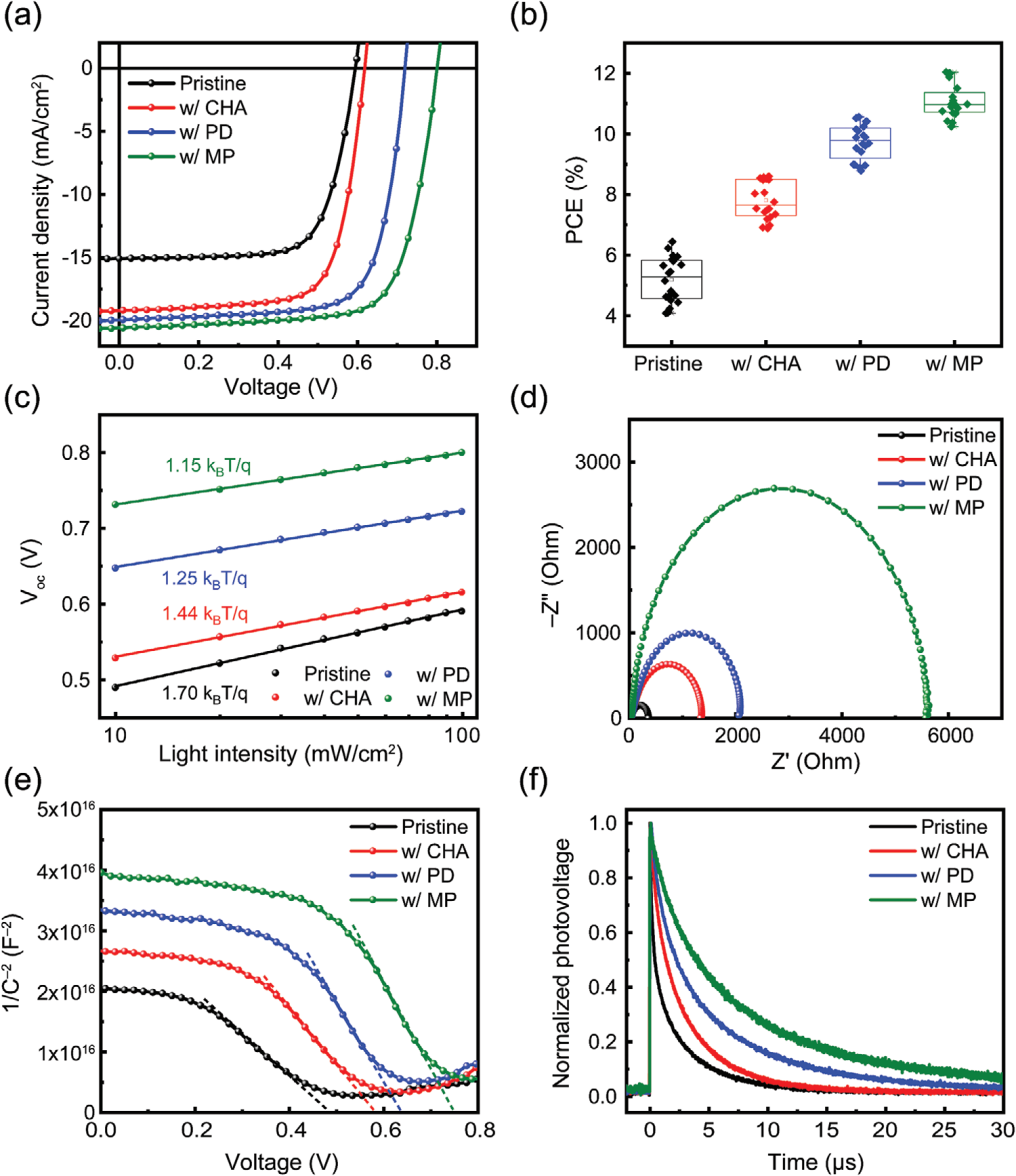
a) *J*–*V* curves of pristine and 2D/3D PSCs. b) PCE distribution of the corresponding devices. c) *V*_oc_ versus light illumination intensity of pristine and 2D/3D PSCs. d) Nyquist plots obtained from EIS of pristine and 2D/3D PSCs. e) Mott–Schottky plots of pristine and 2D/3D PSCs. f) TPV measurements of pristine and 2D/3D PSCs.

To investigate the effect of different organic spacers on device performance, we fabricated PSCs with a structure of ITO (indium tin oxide)/PEDOT: PSS (poly(3,4‐ethylenedioxythiophene): polystyrene sulfonate)/perovskite (without and with different organic spacers)/C_60_/BCP (bathocuproine)/Al. The current density–voltage (*J*–*V*) curves under AM 1.5G 1‐sun illumination, PCE statistics, and the photovoltaic parameters for the best‐performing PSCs are shown (**Figure** **4**a,b; Figure S11, Supporting Information; **Table** **1**). The PCE for the pristine PSCs was 6.44%, with a short‐circuit current density (*J*_sc_) of 15.14 mA cm^−2^, *V*_oc_ of 0.596 V, and a fill factor (FF) of 71.3%. By contrast, the MP‐based PSCs exhibited superior solar cell properties, with a PCE of 12.04%, *J*_sc_ of 20.63 mA cm^−2^, *V*_oc_ of 0.803 V, and a FF of 73.1%. The substantially improved *V*_oc_ of the MP‐based PSCs indicates the substantial suppression of nonradiative recombination in these films. To gain insight into the improvement of photovoltaic parameters of the 2D/3D PSCs, we confirmed the relationship between light intensity and *V*_oc_ of the PSCs (Figure 4c). A deviation of the slope from 1 *k*_B_ *T*/*q* is correlated with trap‐assisted recombination in PSCs, where *k*_B_ is the Boltzmann constant, *T* is the absolute temperature, and *q* is the elementary charge.^[^ ^55^, ^56^ ^]^ The reduced slope value of MP‐based PSCs confirms that nonradiative recombination was remarkably suppressed by incorporating a 2D perovskite into a 3D perovskite. The increase in the peak intensity of steady‐state photoluminescence (PL) of the MP‐based 2D/3D perovskite compared with the peak intensities of its perovskite counterparts also reveals mitigated nonradiative recombination (Figure S12, Supporting Information). Moreover, we conducted the space‐charge‐limited current (SCLC) method to quantify the trap densities in the 3D perovskite and 2D/3D perovskite films. The dark *J*–*V* curves of electron‐only devices with a structure of ITO/SnO_2_/perovskite (without and with different organic spacers)/C_60_/BCP/Al are plotted (Figure S13, Supporting Information). The trap‐filled‐limited voltage (*V*_TFL_) was determined at the kink point where the slope of the full‐logarithmic dark *J*–*V* curves changed from 1 to >3. The *V*_TFL_ is proportional to the defect density (*N*_t_), which can be calculated from the equation

(3) $$N_{t}=\frac{2\varepsilon \varepsilon_{0}V_{TFL}}{qL^{2}}\;$$

where *ε* is the relative dielectric constant, *ε*_0_ is the dielectric constant of vacuum, and *L* is the thickness of the film. The calculated *N*_t_ was 5.00 × 10^16^ cm^−3^ for the electron‐only devices with the 3D pristine perovskite and 3.93 × 10^16^, 3.09 × 10^16^, and 2.87 × 10^16^ cm for the devices with the CHA‐based, PD‐based, and MP‐based 2D/3D perovskites, respectively. These results show that the MP‐based 2D perovskite can efficiently passivate the defect sites at the surface and grain boundary of perovskite, thereby suppressing nonradiative recombination.

**Table 1:** Summary of photovoltaic parameters of champion PSCs with different organic spacers.

	*J*_SC_[mA cm^−2^]	*V*_OC_ [V]	FF [%]	PCE [%]	Average PCE [%]
Pristine	15.14	0.596	71.3	6.44	5.19±0.75
w/ CHA	19.28	0.620	71.9	8.60	7.81±0.63
w/ PD	20.02	0.722	73.0	10.56	9.73±0.58
w/ MP	20.63	0.803	73.1	12.04	11.07±0.55

We also obtained Nyquist plots from electrochemical impedance spectra to investigate the charge transport and recombination dynamics in 3D pristine and 2D/3D PSCs (Figure 4d). The Nyquist plots show a single semicircle; the series resistance (*R*_s_) can be extracted from the high‐frequency region of the arc, and the charge recombination resistance (*R*_rec_) can be obtained from its low‐frequency region.^[^ ^57^, ^58^ ^]^ The *R*_s_ value was higher in the pristine PSCs (*R*_s_ = 74 Ω) than in the CHA‐based (*R*_s_ = 69 Ω), PD‐based (*R*_s_ = 53 Ω), and MP‐based PSCs (*R*_s_ = 44 Ω). By contrast, the *R*_rec_ value was lower in the pristine PSCs (*R*_rec_ = 360 Ω) than in the CHA‐based (*R*_rec_ = 1340 Ω), PD‐based (*R*_rec_ = 2060 Ω), and MP‐based (*R*_rec_ = 5570 Ω) PSCs. Notably, the MP‐based PSCs had a lower *R*_s_ value and a substantially higher *R*_rec_ value than all of the other PSCs, indicating enhanced charge transport and suppressed charge recombination in the MP‐based 2D/3D heterostructure. Moreover, the dark *J*–*V* curves of PSCs show a reduced leakage current with a low *R*_s_ in the MP‐based PSCs (Figure S14, Supporting Information); these results are consistent with the Nyquist plots.

To further study the mechanisms of improved photovoltaic properties in the 2D/3D PSCs, we obtained the capacitance^–2^ – voltage (*C* ^−2^–*V*) curves of the pristine and 2D/3D PSCs (Figure 4e) and conducted Mott–Schottky analysis to extract the built‐in potential (*V*_bi_), which is strongly related to the *V*_oc._^[^ ^30^ ^]^ The *V*_bi_ value can be obtained from the intercept of the x‐axis with the line extrapolated from the linear‐drop regime of the *C* ^−2^–*V* plot. The extracted *V*_bi_ value was 0.48 V for the pristine PSC and 0.58, 0.64, and 0.75 V for the CHA‐based, PD‐based, and MP‐based PSCs, respectively. The higher value of *V*_bi_ in the 2D/3D PSCs relative to that in the pristine PSCs indicates that the 2D/3D heterostructure enhances the driving force for charge extraction and reduces the recombination losses at the interface between perovskite and the electron‐transport layer.^[^ ^59^ ^]^ Moreover, the interfacial charge density is inversely related to the slope of the *C* ^–2^–*V* plot in the linear‐drop regime. The charge density was lower in the 2D/3D PSCs than in the pristine PSCs. This difference indicates that charge transfer at the contact interface is faster in the 2D/3D PSCs than in the pristine PSCs.^[^ ^60^ ^]^ These results are consistent with the trends of the improvements in the *J*_sc_ and *V*_oc_ values of the 2D/3D PSCs.

This could be further verified from the energy level diagrams of the PSCs determined from the ultraviolet photoelectron spectroscopy (UPS) (Figure S15, Supporting Information), and optical bandgap extracted from the Tauc plot (Figure S8b, Supporting Information). The reduced energy difference between the conduction band minimum (CBM) of the MP‐based 2D/3D perovskite and the lowest unoccupied molecular orbital (LUMO) of the electron transport layer induces the energetically favored band alignment resulting in the increased *V*_oc_ and electron extraction. We also further confirmed the charge transport dynamics using transient photovoltage (TPV) and transient photocurrent (TPC) measurements (Figure 4f; Figure S16, Supporting Information). The average charge recombination lifetime (τ_rec_) and charge extraction lifetime (τ_ext_) can be extracted by fitting TPV and TPC curves to a biexponential decay function, respectively. The value of *τ*_rec_ was higher for the MP‐based PSC (*τ*_rec_ = 7.50 µs) than for the PD‐based (*τ*_rec_ = 4.80 µs), CHA‐based (*τ*_rec_ = 2.63 µs), and pristine (*τ*_rec_ = 1.65 µs) PSCs. By contrast, *τ*_ext_ was lower for the MP‐based PSC (*τ*_ext_ = 0.73 µs) than for the PD‐based (*τ*_ext_ = 1.33 µs), CHA‐based (*τ*_ext_ = 1.76 µs), and pristine (*τ*_ext_ = 1.90 µs) PSCs. The MP‐based PSCs showed the fastest charge extraction and the lowest charge recombination among the investigated PSCs, which is consistent with the aforementioned electrical analysis.

**Figure 5:**
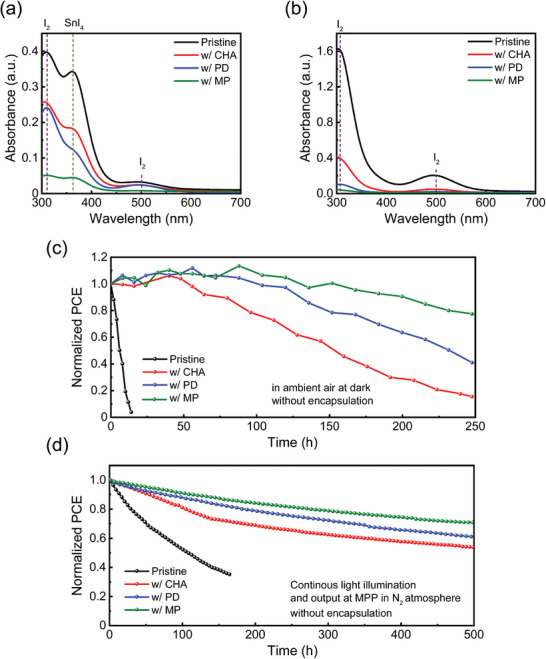
a) UV–vis absorption spectra of degradation products dissolved in toluene from 3D and 2D/3D perovskite films after exposure to air for 6 h in the dark. b) UV–vis absorption spectra of degradation products dissolved in toluene from 3D and 2D/3D perovskite films under 1‐sun light illumination for 6 h. The procedure was performed in a glovebox to minimize the generation of SnI_4_. c) Normalized PCEs versus storage times of pristine and 2D/3D PSCs without encapsulation under a dark condition in ambient air (20–25 °C, relative humidity 35–40%). d) Normalized PCEs versus storage times of devices without encapsulation under continuous illumination at a fixed voltage near the MPP under the N_2_ atmosphere.

We also investigated the stability of the 3D pristine and 2D/3D perovskite films and the associated PSCs under various test conditions. After exposure to air for 6 h in the dark to promote the formation of SnI_4_, the 3D pristine and 2D/3D perovskite films were immersed in toluene (Figure S17a, Supporting Information); the UV–vis absorption spectra of solution were then obtained for analysis of the dissolved degradation products (**Figure** **5**a). All of the spectra show a shoulder peak at ≈360 nm,^[^ ^17^ ^]^ which indicates that the SnI_4_ is formed as a result of perovskite film degradation in ambient air. The intensity of the corresponding peak decreased in the order of 3D pristine, CHA‐based, PD‐based, and MP‐based 2D/3D perovskite films. All of the spectra had two peaks at ≈300 and ≈500 nm, which are both assigned to I_2_,^[^ ^17^ ^]^ which likely formed by the degradation of SnI_4_ due to the inevitable light exposure during sample preparation and measurements.^[^ ^61^ ^]^ In addition, the 3D pristine and 2D/3D perovskite films were immersed in toluene and then aged for 6 h under light illumination. This procedure was conducted in a glovebox to minimize the formation of SnI_4_ (Figure S17b, Supporting Information). The UV–vis absorption spectra of the dissolved degradation products in toluene were obtained (Figure 5b). The spectrum of the 3D pristine shows a prominent absorption peak of I_2_ by facile reaction of I^–^ and a hole under illumination; this peak is less pronounced in the spectra of the 2D/3D perovskites and is notably absent in the spectrum of the MP‐based 2D/3D perovskite. These results demonstrate that the MP‐based 2D perovskite phase efficiently inhibits the formation of SnI_4_ and I_2_ in the 2D/3D heterostructure. This stabilized framework of heterostructure is attributed to strengthened hydrogen bonding and intermolecular interaction within the 2D perovskite. These results are consistent with the decreased *E*_f_ of neutral Sn and I vacancies (V_Sn_ and V_I_).

Finally, we tested the stability of the pristine and 2D/3D PSCs using the International Summit on Organic Photovoltaic Stability (ISOS) protocol.^[^ ^62^ ^]^ We used the ISOS‐D‐1 protocol to monitor the degradation of the PCEs of the unencapsulated devices in the dark in ambient air (20–25 °C, 35–40 RH%) (Figure 5c). The pristine PSCs were almost fully degraded after 14 h, whereas the MP‐based PSCs showed superior stability, retaining 77% of their initial PCE after 250 h. To further confirm the hydrophobic nature of the 2D/3D perovskite films, we measured the water contact angles of the 3D perovskite and 2D/3D perovskite films (Figure S18, Supporting Information). The contact angle was smaller for the 3D pristine film (44.4°) than for the CHA‐based (53.8°), PD‐based (72.6°), and MP‐based (79.9°) 2D/3D perovskite films. This increase in water contact angle indicates that the 2D/3D perovskite films became more hydrophobic than the 3D perovskite films. Moreover, we used the ISOS‐L‐1I protocol to evaluate the stability of the pristine and 2D/3D PSCs without encapsulation under continuous light illumination at a fixed voltage near the MPP under an N_2_ atmosphere (Figure 5d). The pristine PSCs retained 35% of their initial PCE after 166 h, whereas the MP‐based PSCs retained 71% of their initial PCE after 500 h. The robust ambient and operational stability of the MP‐based PSCs is attributed to the hydrophobic nature of the 2D perovskite phase and to defect passivation due to the inorganic framework stabilized by tailored interaction within the 2D perovskite phase in a 2D/3D heterostructure.

## 3 Conclusion

In summary, we have demonstrated dimensionality engineering of Sn‐based perovskite using an MP^+^ cation, to improve both the efficiency and stability of PSCs. Incorporating a secondary ammonium and ether group into CHA has enhanced the rigidity of 2D perovskite, which results in strengthening the hydrogen bonding between the [SnI_6_]^4–^ framework and the organic spacer molecules. Furthermore, the ether group of the organic spacers induces weak C─H─O hydrogen bonding between the organic spacer molecules and thereby enhances their intermolecular interaction. The strong hydrogen bonding and intermolecular interaction among the 2D/3D perovskite crystals lead to strong out‐of‐plane preferential growth and to decreased structural and energetical disorder. The structurally stabilized 2D/3D perovskite facilitates charge transport at the interface while suppressing the nonradiative recombination and generation of degradation products. Consequently, the MP‐based PSC achieved the highest PCE of 12.04%. The superior oxidative and operational stability of the MP‐based PSC indicates that the 2D perovskite structure is robust as a result of the synergetic effect of hydrogen bonding and intermolecular interaction. We believe our work paves a new way for using molecularly engineered organic spacers to improve the efficiency and stability of Sn‐based PSCs through the design of 2D/3D heterostructures.

## Conflict of Interest

The authors declare no conflict of interest.

## Supporting information

Supporting Informatio

## Data Availability

Research data are not shared.
