# Supplementary material for: Molecularly Engineered Alicyclic Organic Spacers for 2D/3D Hybrid Tin‐based Perovskite Solar Cells
Source: Small. 2024 Sep 3;20(48):2405598. doi: 10.1002/smll.202405598 (PMC11600702; doi:10.1002/smll.202405598)
Supplement: Supplementary file 1 — Supporting Informatio [file SMLL-20-2405598-s001.docx]

**Supporting Information**

**Molecularly Engineered Alicyclic Organic Spacers for 2D/3D Hybrid Tin-based Perovskite Solar Cells**

Jinhyeok Choi, Jimin Kim, Minyoung Jeong, Byeongchan Park, Seunghyun Kim, Jisang Park, and Kilwon Cho*

J. Choi, J. Kim, M. Jeong, B. Park, S. Kim, J. Park, and Prof. K. Cho

Department of Chemical Engineering

Pohang University of Science and Technology

Pohang, 37673, Korea

* Corresponding author: Prof. K. Cho (kwcho@postech.ac.kr)

**Experimental Section**

***Materials*:** PEDOT:PSS water dispersion (Clevios VP Al 4083) was purchased from Heraeus. Sn (99.99%), I_2_ (>99.8%), SnF_2_ (99%), C_60_ (>99.9%), bathocuproine (BCP; 99.99%), N, N-dimethylformamide (DMF, 99.8%, anhydrous), dimethyl sulfoxide (DMSO, 99.8%, anhydrous), diethyl ether (>99.7%, anhydrous) were purchased from Sigma-Aldrich. Formamidinium iodide (FAI), cyclohexylammonium iodide (CHAI), piperidinium iodide (PDI), and morpholinium iodide (MPI) were purchased from GreatCell Solar. SnO_2_ (15 wt% in H_2_O) was purchased from Alfa Aesar.

***Solar cell fabrication*:** ITO glasses were cleaned sequentially in detergent aqueous solution, deionized water, acetone, and isopropanol, each with ultrasonication for 30 min. The cleaned substrates were treated with UV-ozone for 30 min. PEDOT:PSS was spin-coated onto ITO glass at 4000 rpm for 60 s, then dried at 120 °C for 30 min. Afterward, samples were transferred to a nitrogen glovebox. A 0.8 M SnI_2_ solution was prepared as follows. 0.6 mmol of I_2_ and an excess of 1 mmol of Sn were added to 7.5 ml of mixed solvent (DMF: DMSO = 4:1, v/v), and stirred overnight. A 0.8 M FASnI_3_ perovskite precursor solution comprised of FAI, synthesized SnI_2_ solution, and SnF_2_ in the molar ratio of 1:1:0.1 was prepared and stirred at room temperature for 3h. A 0.8 M 2D perovskite (CHA_2_SnI_4_, PD_2_SnI_4_, and MP_2_SnI_4_) precursor solution comprised of ammonium halide salts, synthesized SnI_2_ solution, and SnF_2_ in the molar ratio of 2:1:0.1 was prepared and stirred at room temperature for 3h. For the 2D/3D hybrid perovskite solution, 5 mol% of the 2D perovskite precursor and 95 mol% of the 3D perovskite precursor were mixed. Each precursor solution was filtered with a 0.2 μm PTFE filter, and then spin-coated onto the substrate at 1000 rpm for 10 s, then 5000 rpm for 30 s. Diethyl ether was used as the anti-solvent during the spin coating process. The perovskite film was annealed at 70 °C for 20 min. Then 30-nm-thick C_60_, 6-nm-thick BCP, and 100-nm-thick Al were sequentially deposited with thermal evaporation under vacuum < 10^-6^ Torr.

Electron-only devices were fabricated as follows. SnO_2_ solution diluted with DI water (SnO_2_: DI water=1:3, v/v) was filtered with a 0.2 μm PVDF filter and then spin-coated onto the ITO glasses at 3000 rpm for 30 s. The SnO_2_ film was annealed at 180 °C for 20 min. Perovskite was deposited on the substrates, and 30-nm-thick C_60_, 6-nm-thick BCP, and 100-nm-thick Al were sequentially deposited with thermal evaporation under vacuum < 10^-6^ Torr.

***Solar cell measurements*:** *J-V* measurements were conducted in a N_2_-filled glove box under AM 1.5G illumination with an intensity of 100 mW cm^-2^ by using a Keithley 2400 and an Oriel 1 kW solar simulator referenced to a Reference Cell PVM 132 calibrated at the US National Renewable Energy Laboratory. A mask with an area of 0.0555 cm^2^ was used for the electrode deposition. EIS and Mott–Schottky analysis were conducted in the dark by using an MFIA Impedance Analyzer. TPV and TPC measurements were performed in a homemade system that uses a laser with a wavelength of 536 nm for pulsed excitation under white light. The stability test at the fixed voltage near the maximum power point (MPP) was performed as follows. A fixed voltage corresponding to the voltage at MPP was applied during the stability test, and photocurrent at MPP was monitored over time.

***Characterization*:** XPS and UPS spectra were recorded by using a Thermo Scientific ESCALAB 250Xi. XRD and GIXRD patterns were obtained with a Rigaku D/Max-2500 diffractometer with Cu-Kα X-rays. GIWAXS measurements were performed using the synchrotron source at the 3C and 9A beamlines, and XANES measurements were performed using the synchrotron source at the 4D and 10A2 beamlines at the Pohang Accelerator Laboratory (PAL) in Korea. The SEM images were obtained with a Hitachi S-4800. Steady-state PL measurements were conducted by Horiba Jobin Yvon Nanolog. The absorption spectra were measured using a Perkin Elmer Lambda 1050. The liquid-state ^1^H NMR spectra were collected by using a Bruker Avance 400 spectrometer. FT-IR spectra were obtained by Bruker VERTEX 70 under vacuum condition. The TOF-SIMS depth profiles were obtained with an Ion-tof TOF-SIMS 5-100.

***Computational methods*:** DFT calculations were performed at the generalized gradient approximation (GGA) level in the Perdew−Burke−Ernzerhof (PBE) exchange–correlation functional as implemented in CASTEP from Materials Studio 2020. The electron-ion interactions were described by using ultrasoft pseudopotentials generated on-the-fly with electrons from H 1s; the C, N, and O 2s, 2p; I 5s, 5p; and Sn 4d, 5s, 5p shells were included in the calculations. Dispersion correction to the PBE functional was done within the method of Tkatchenko and Scheffler (DFT-TS) to take into account the van der Waals interactions. All geometry structures were fully relaxed through geometric optimization. An energy cutoff of 630 eV and 1 × 1 × 1 k-point meshes for the structures were used to achieve an energy of 1 × 10^−5^ eV/atom, a force of 0.03 eV/Å, and a displacement convergence of 0.001 Å. The formation energies were calculated as follows:

$${FE}_{2D}=E\left( L_{2}SnI_{4} \right)-E\left( SnI_{2} \right)-2E\left( LI \right), (L=CHA, PD, MP)$$

$${FE}_{3D}=E\left( FASnI_{3} \right)-E\left( SnI_{2} \right)-E\left( FAI \right)$$

where ${FE}_{2D}$ and ${FE}_{3D}$ are the formation energy of 2D and 3D perovskite cells, and $E\left( L_{2}SnI_{4} \right)$ and $E\left( FASnI_{3} \right)$are the total energy of 2D and 3D perovskite cells. $E\left( SnI_{2} \right)$ is the energy of SnI_2_ that is taken to be that of bulk. $E\left( LI \right)$ and $E\left( FAI \right)$ are the energy of ammonium salts that are calculated under the assumption that they do not interact with each other.

The formation energy of neutral vacancy (${FE}_{vacancy}$) was calculated as follows:

$${FE}_{vacancy}=E\left( PVK_{vacancy} \right)+\mu-E(PVK_{ideal})$$

where $E\left( PVK_{vacancy} \right)$ and $E\left( PVK_{ideal} \right)$ are the total energy of defective and ideal perovskite cells, and the $\mu$ is the chemical potential of Sn and I.


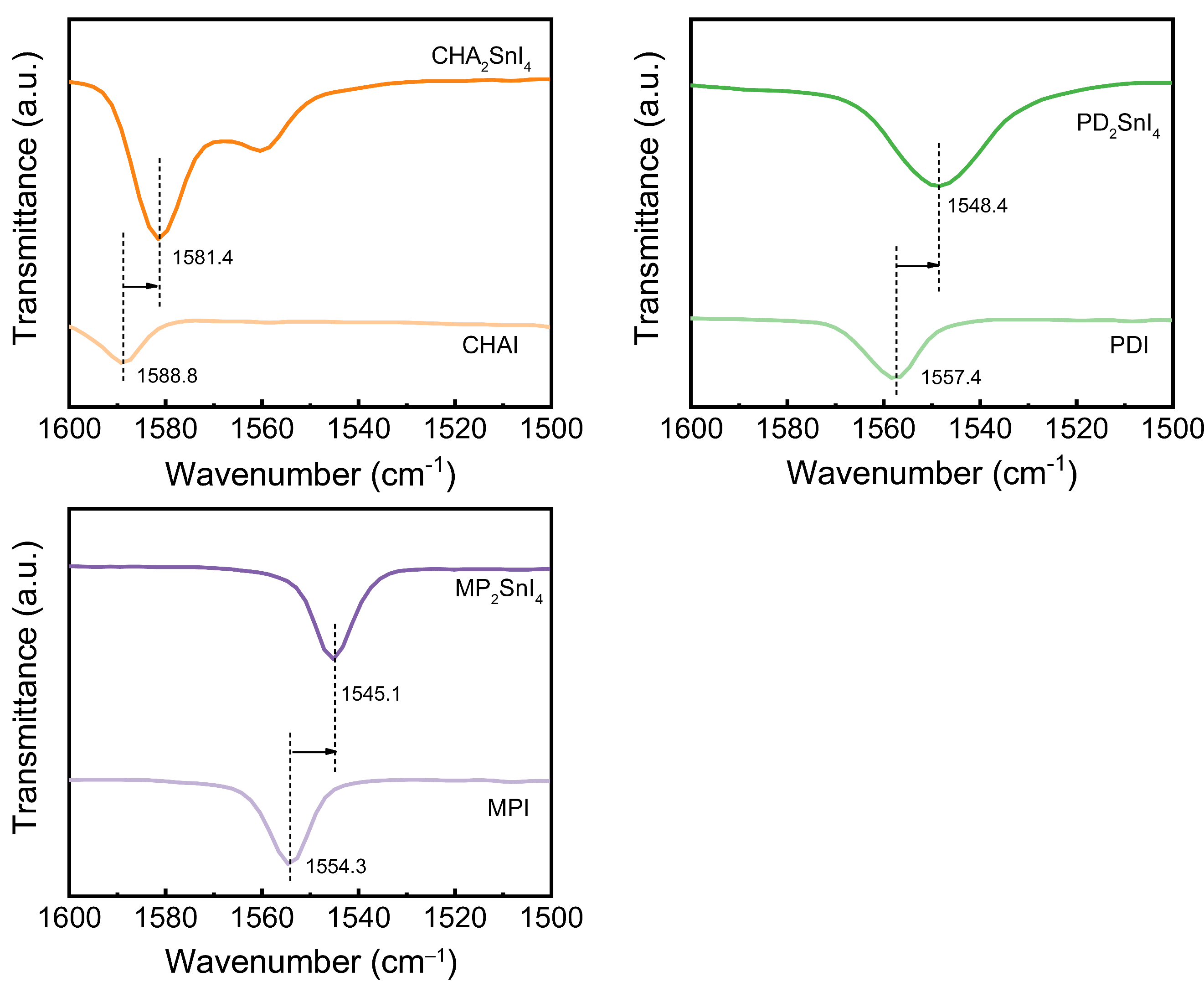


**Figure S1**. FT-IR spectra of the films made of either pure organic spacers or 2D perovskite.


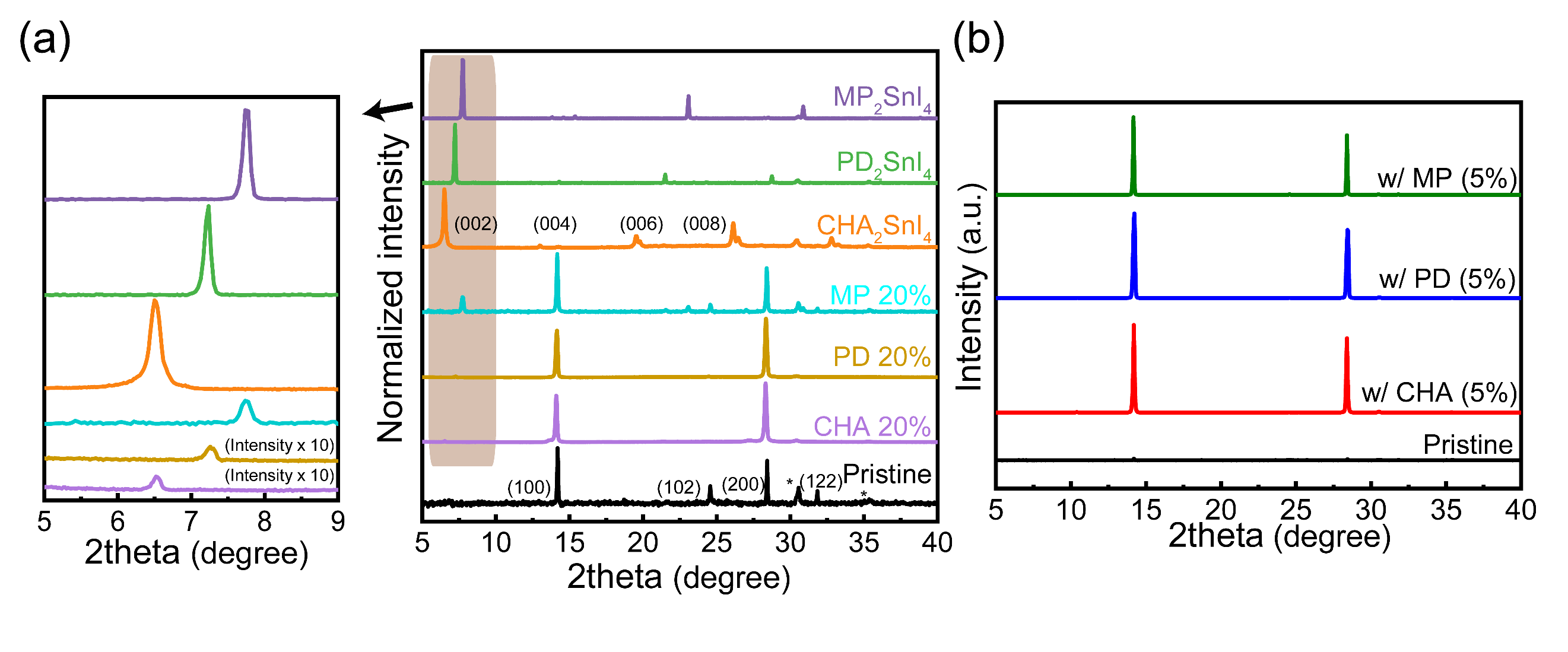


**Figure S2.** (a) Normalized XRD patterns of 3D, 2D/3D perovskite with 20 mol% of organic spacers, and pure 2D perovskite (*is indexed to ITO). Left: Enlarged patterns of corresponding films at a low angle (2*θ* < 10°). (b) XRD patterns of 3D and 2D/3D perovskite films with optimum concentration of organic spacers (5 mol%).

**
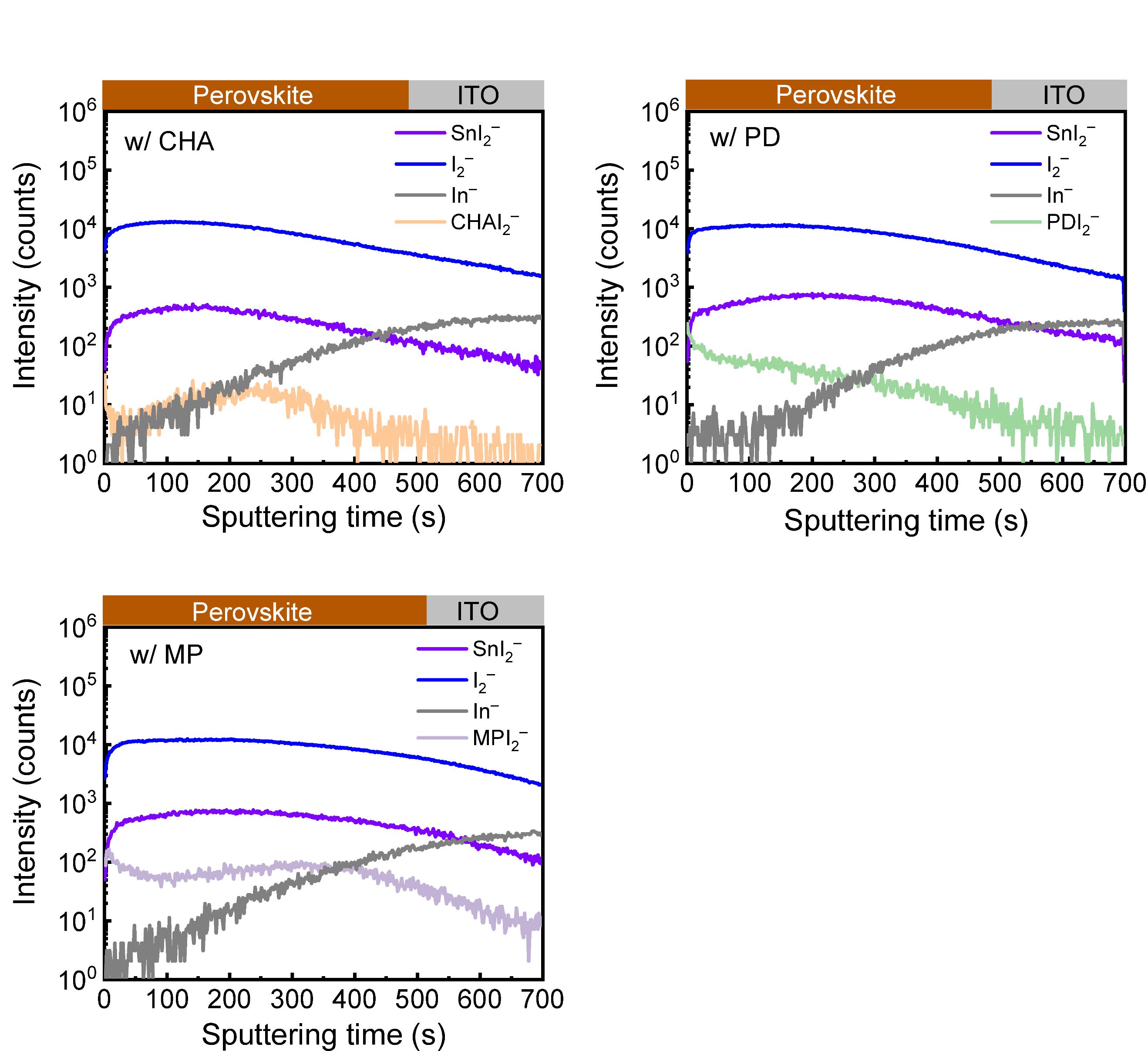
**

**Figure S3**. TOF-SIMS of 2D/3D perovskite films.


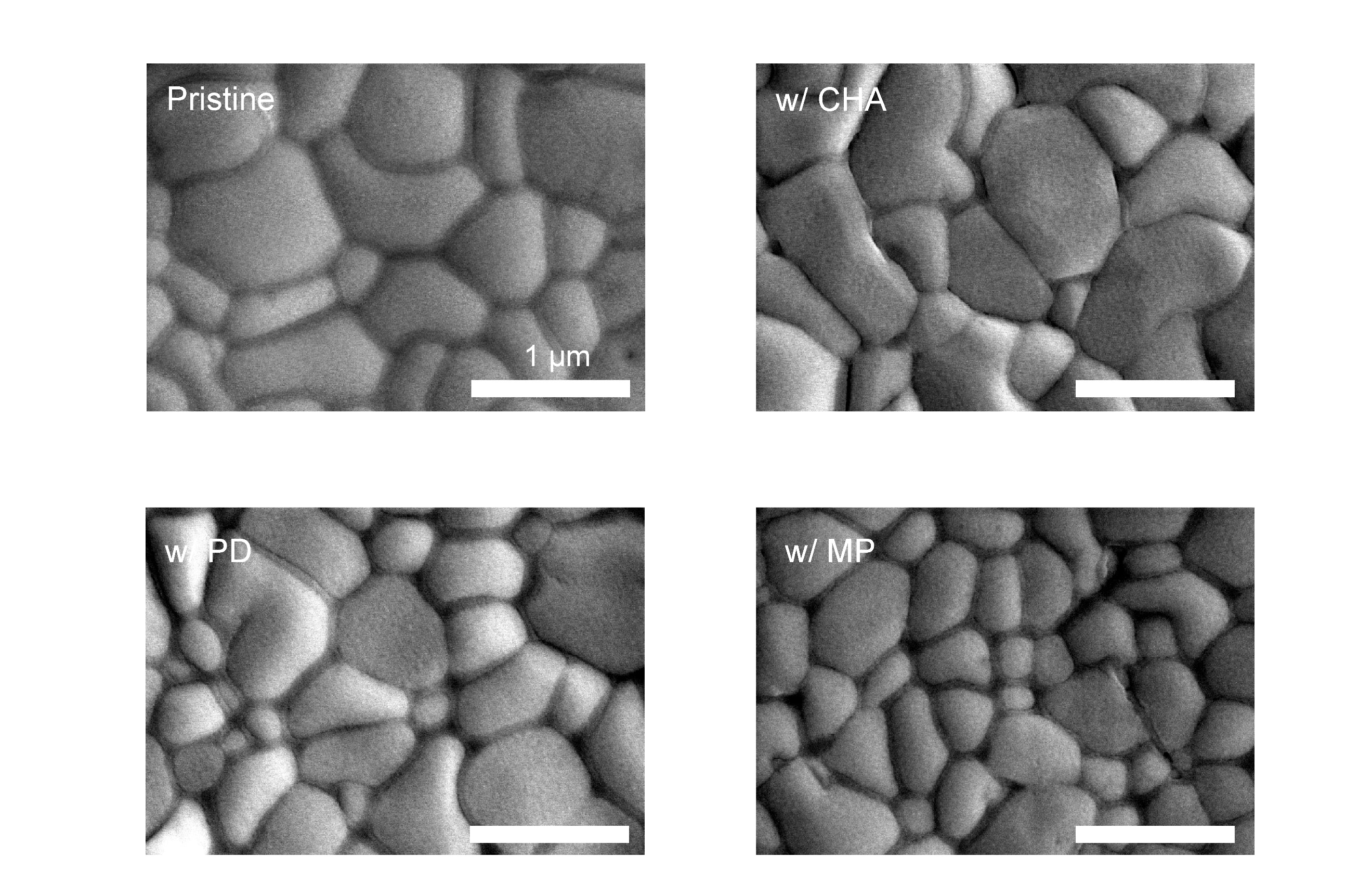


**Figure S4.** SEM images of 3D and 2D/3D perovskite films.


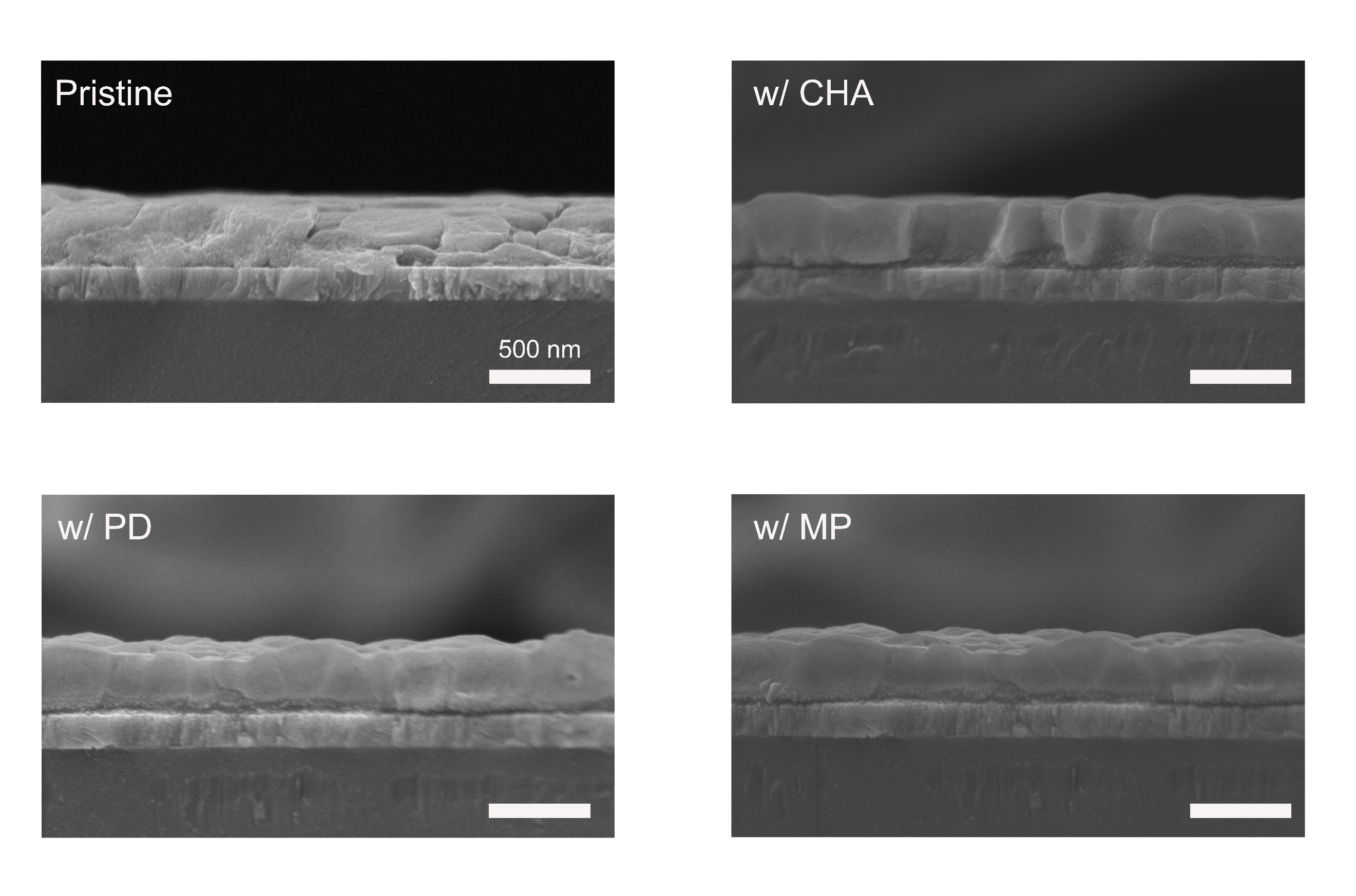


**Figure S5.** Cross-sectional SEM images of 3D and 2D/3D perovskite films.


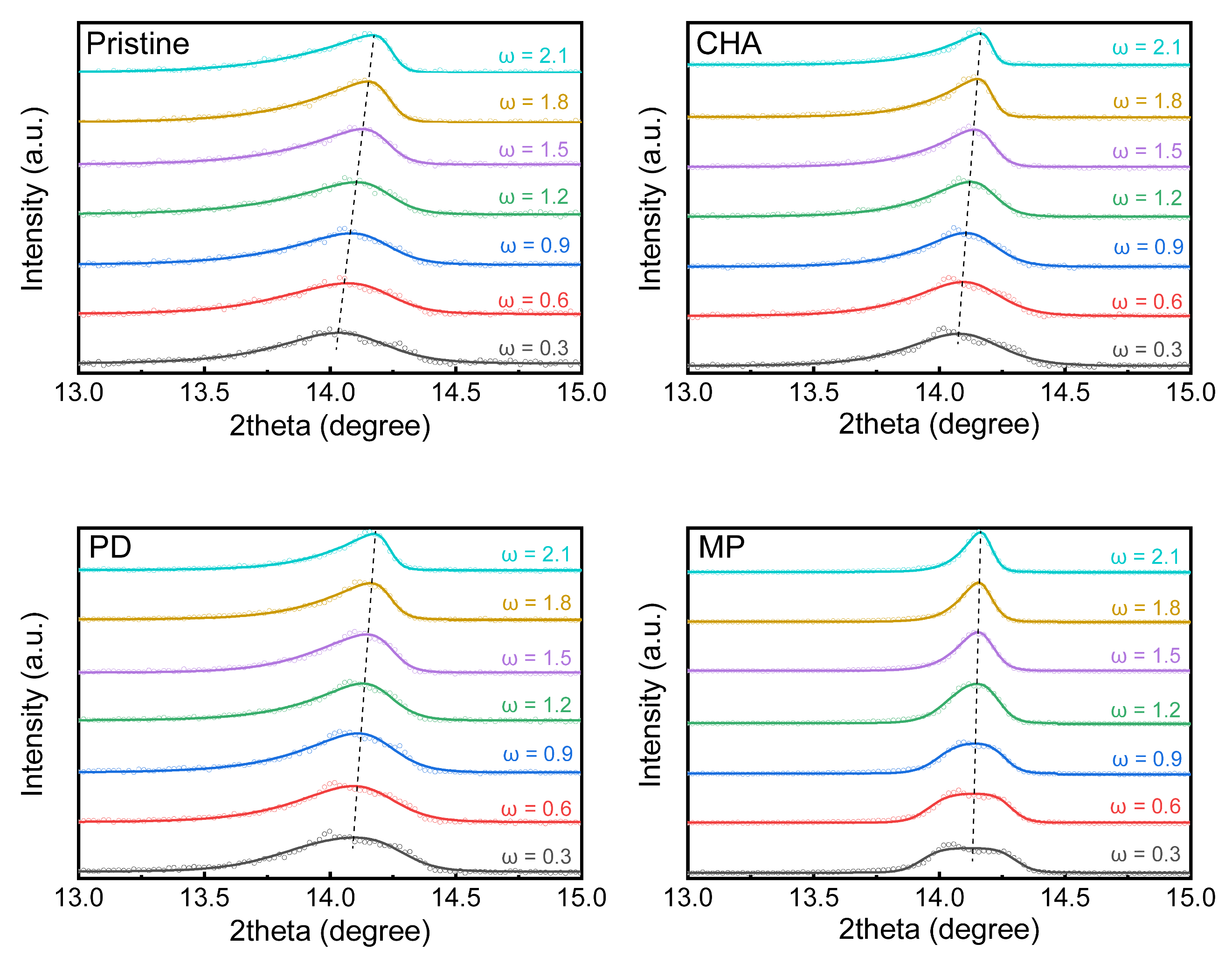


**Figure S6**. GIXRD spectra of 3D and 2D/3D perovskite film measured at different grazing incident angle (ω).


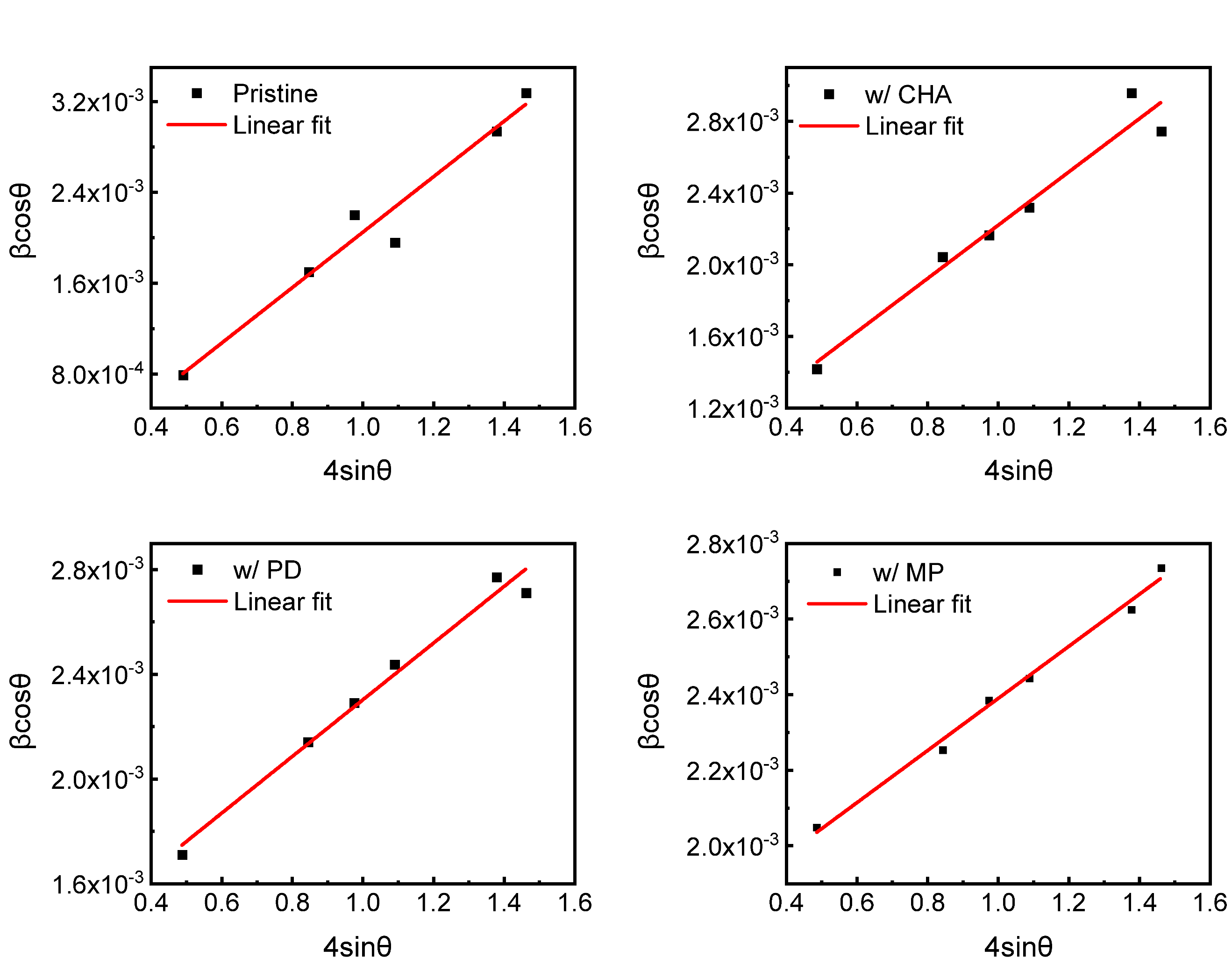


**Figure S7**. Williamson–Hall plots of 3D and 2D/3D perovskite films. The slope of the fitting line is the microstrain.


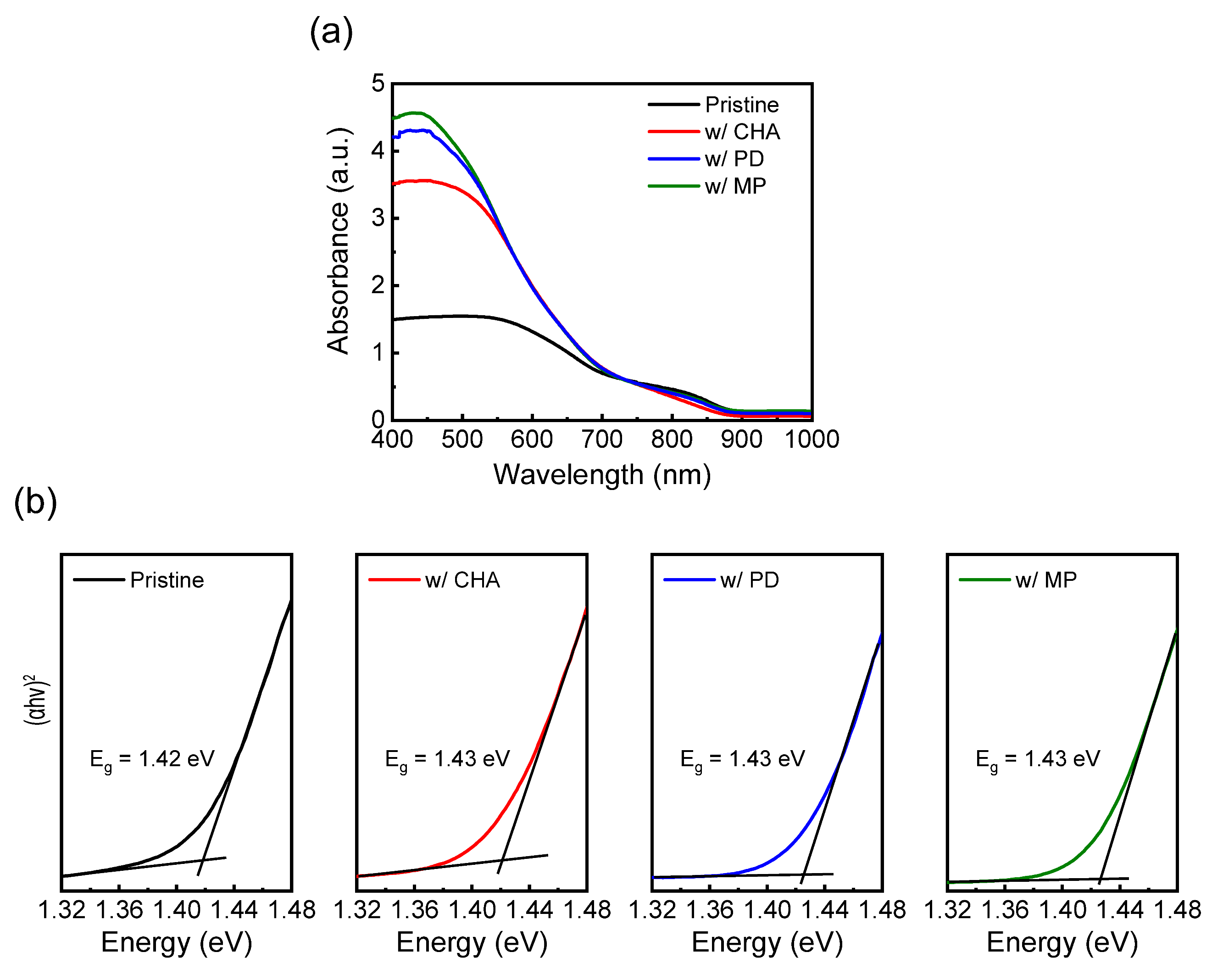


**Figure S8**. (a) Absorption spectra and (b) Tauc plots of the 3D and 2D/3D perovskite films.


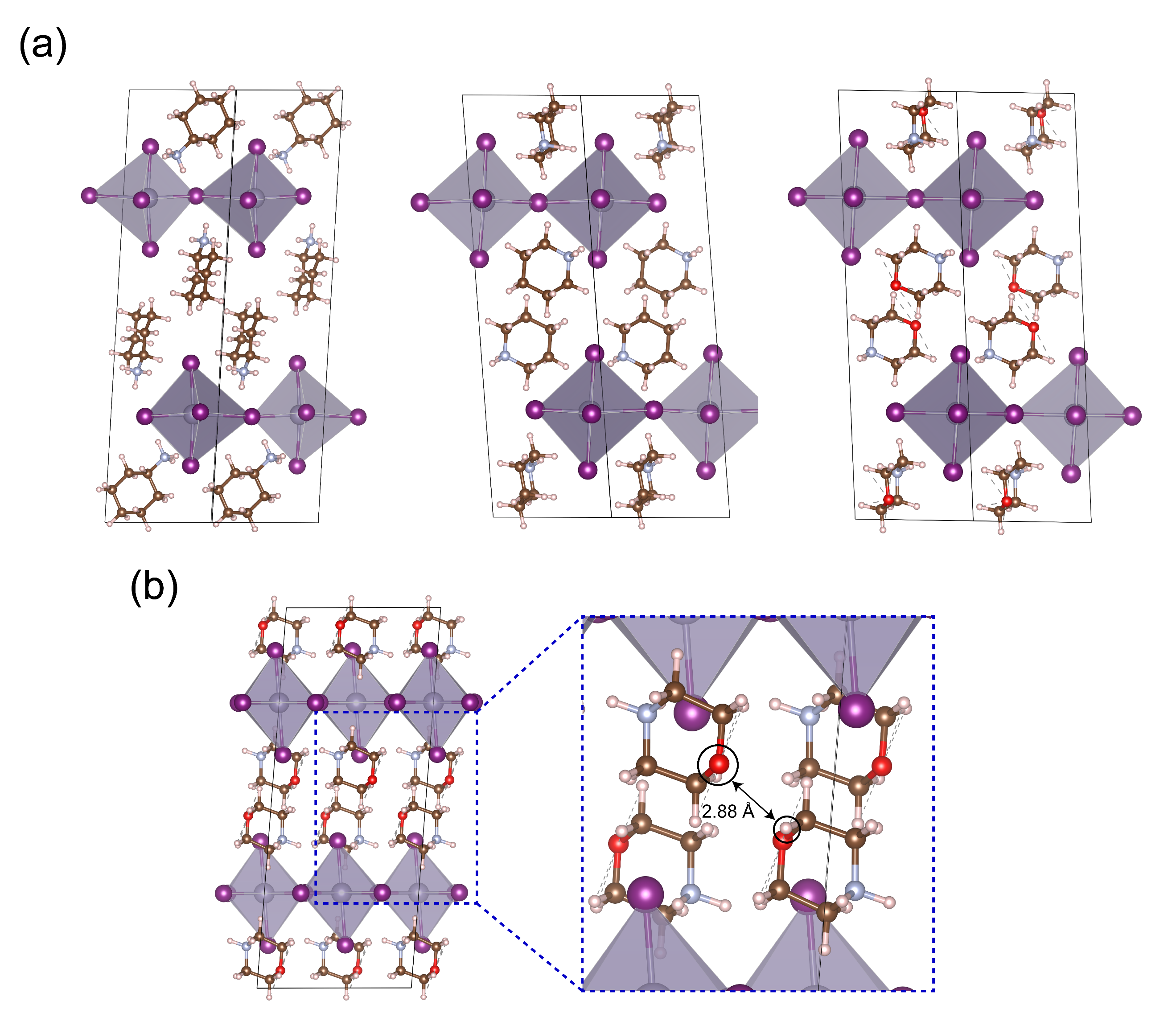


**Figure S9**. (a) Structural model of CHA_2_SnI_4_, PD_2_SnI_4_, and MP_2_SnI_4_. All structures were geometrically fully relaxed by DFT calculation. (b) Side view of the MP_2_SnI_4_ structure. Enlarged structure shows the shortest distance between the O atom of one MP^+^ and the H atom of the C–H of another MP^+^.


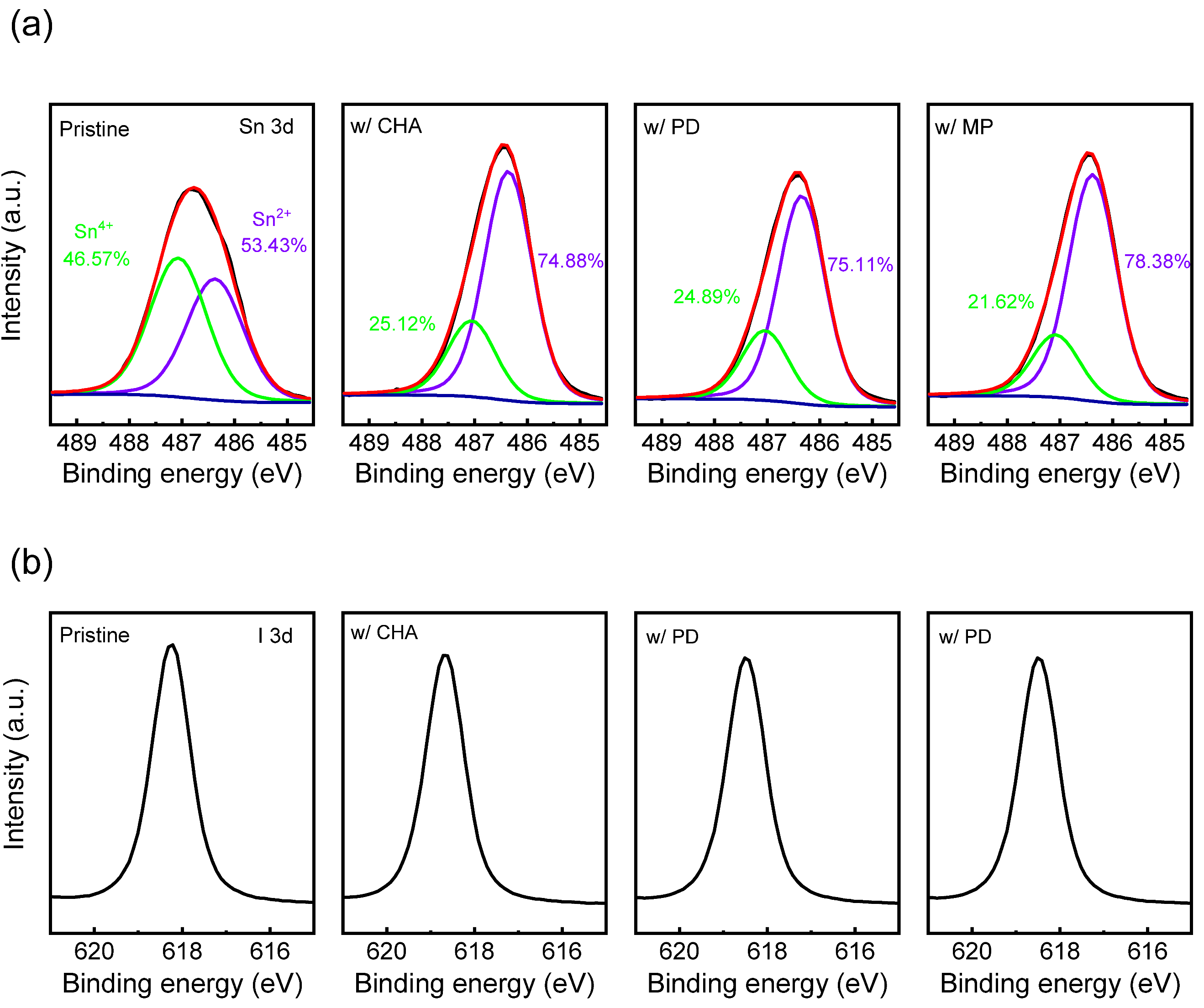


**Figure S10.** XPS spectra of (a) Sn 3*d* and (b) I 3*d* peak of 3D and 2D/3D perovskite films.


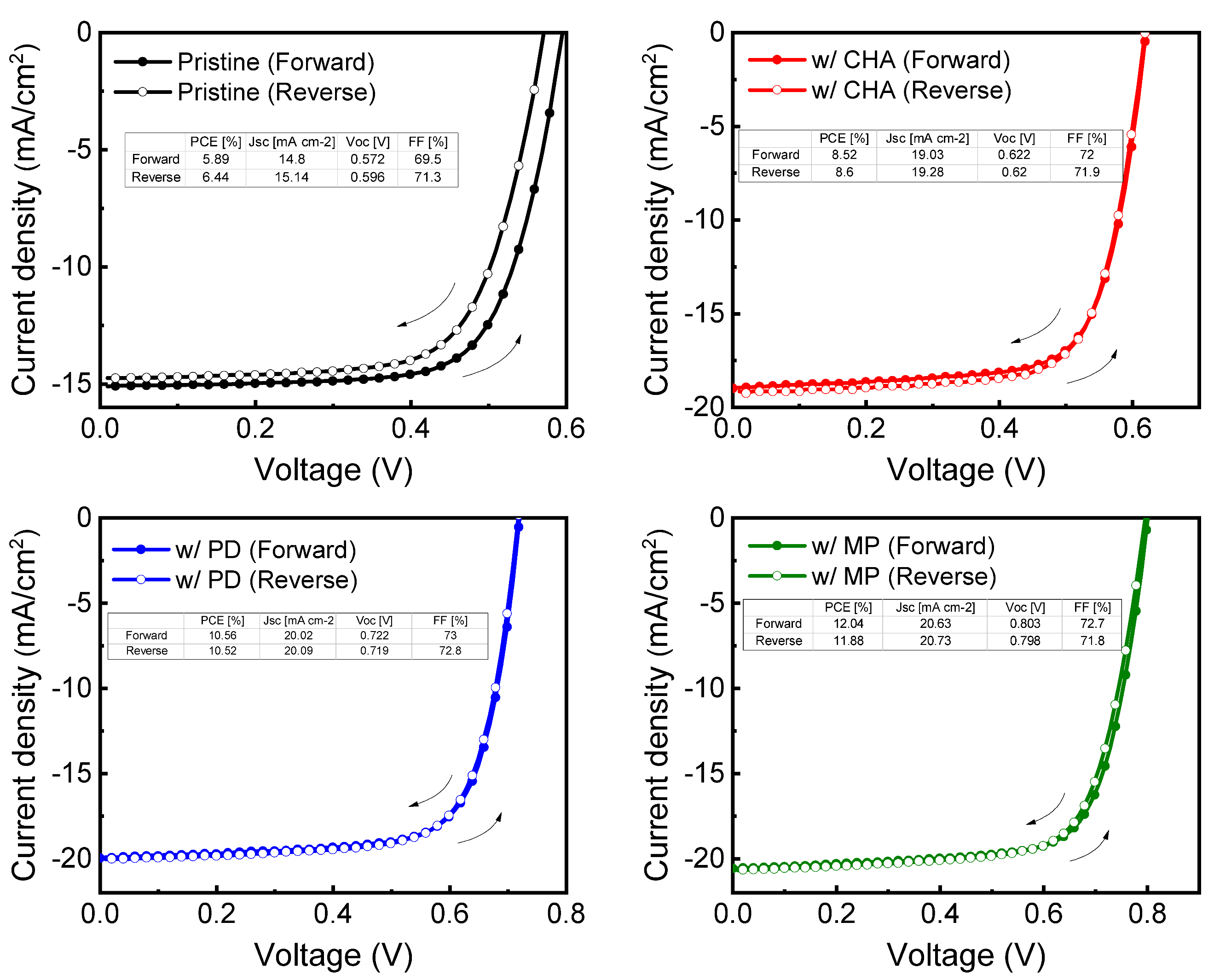


**Figure S11**. *J*–*V* curves of pristine and 2D/3D PSCs under 1-sun illumination with different can direction


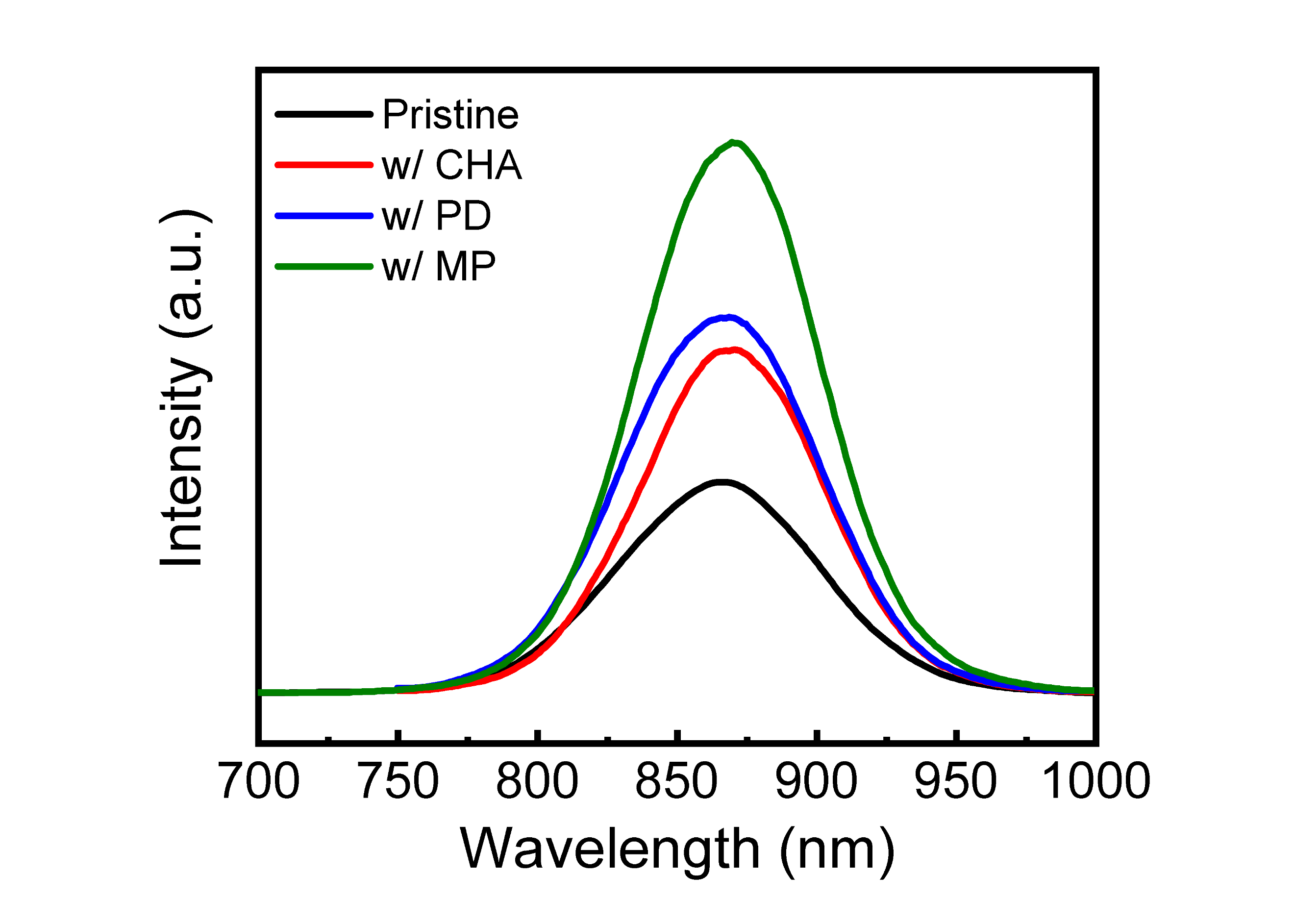


**Figure S12**. Steady-state PL spectra of 3D and 2D/3D perovskite films.


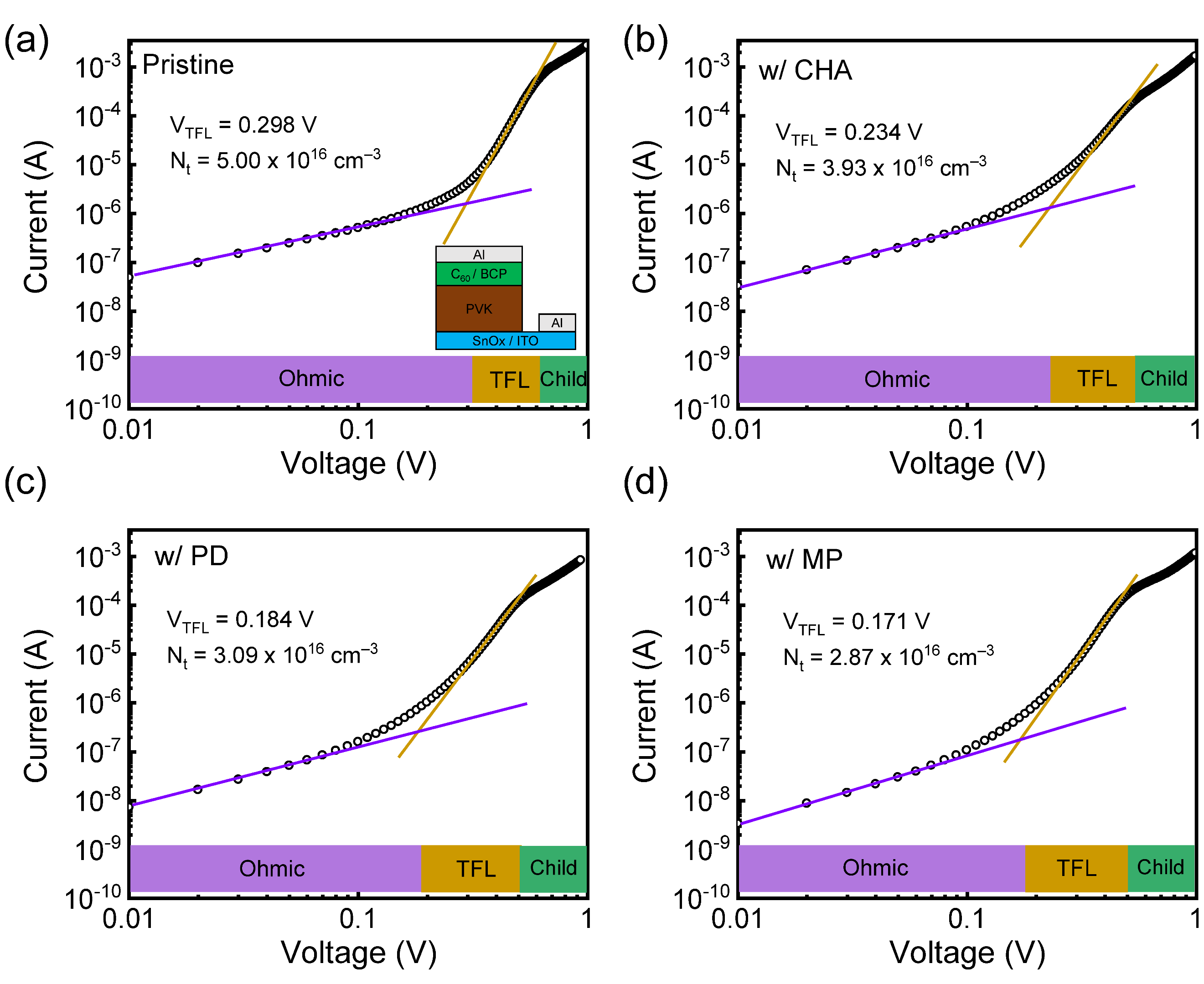


**Figure S13**. SCLC method of electron-only devices with a structure of ITO/SnO_2_/Perovskite (without and with different organic spacers)/C_60_/BCP/Al.


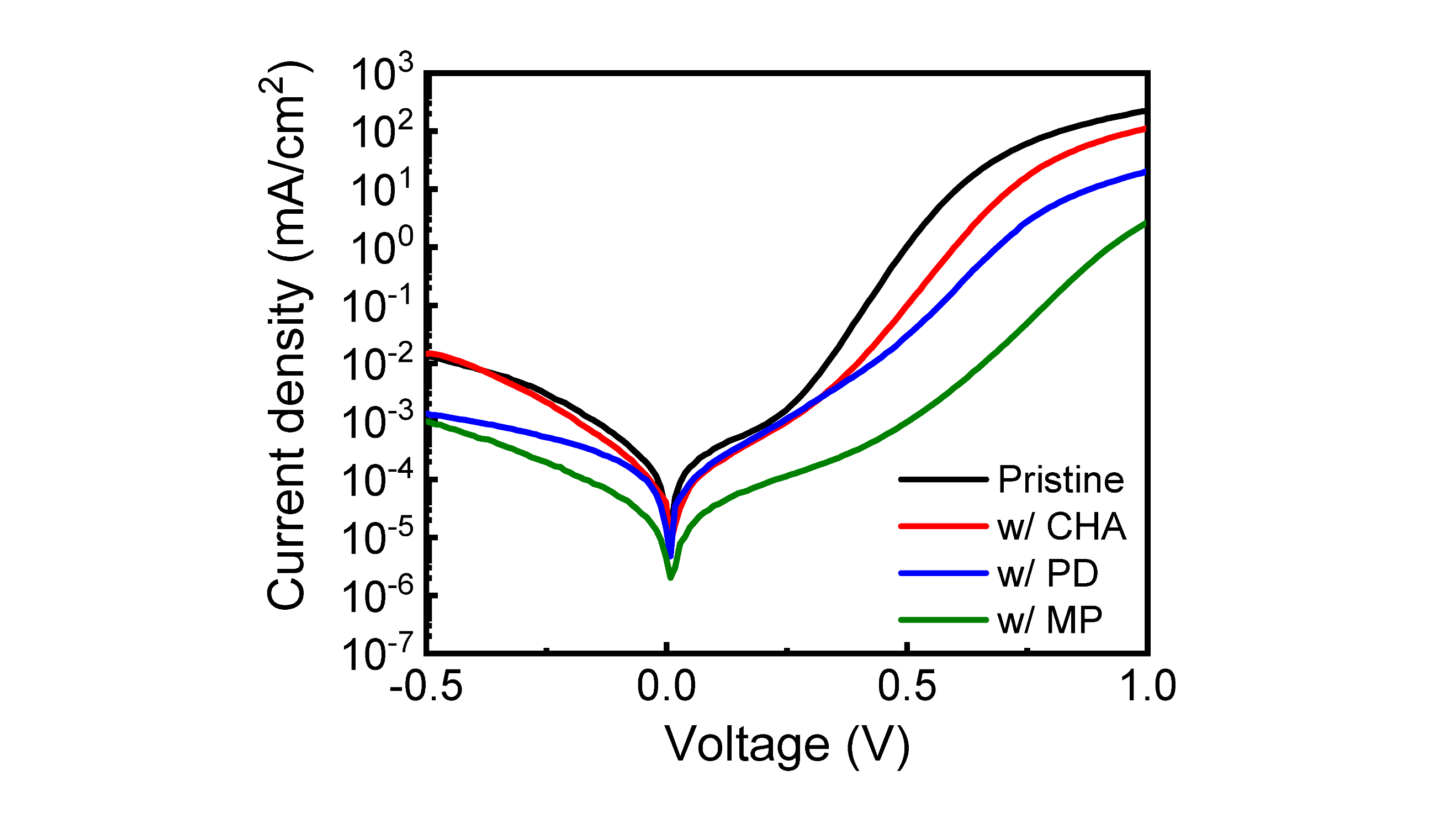


**Figure S14**. Dark *J*–*V* plots of pristine and 2D/3D PSCs.


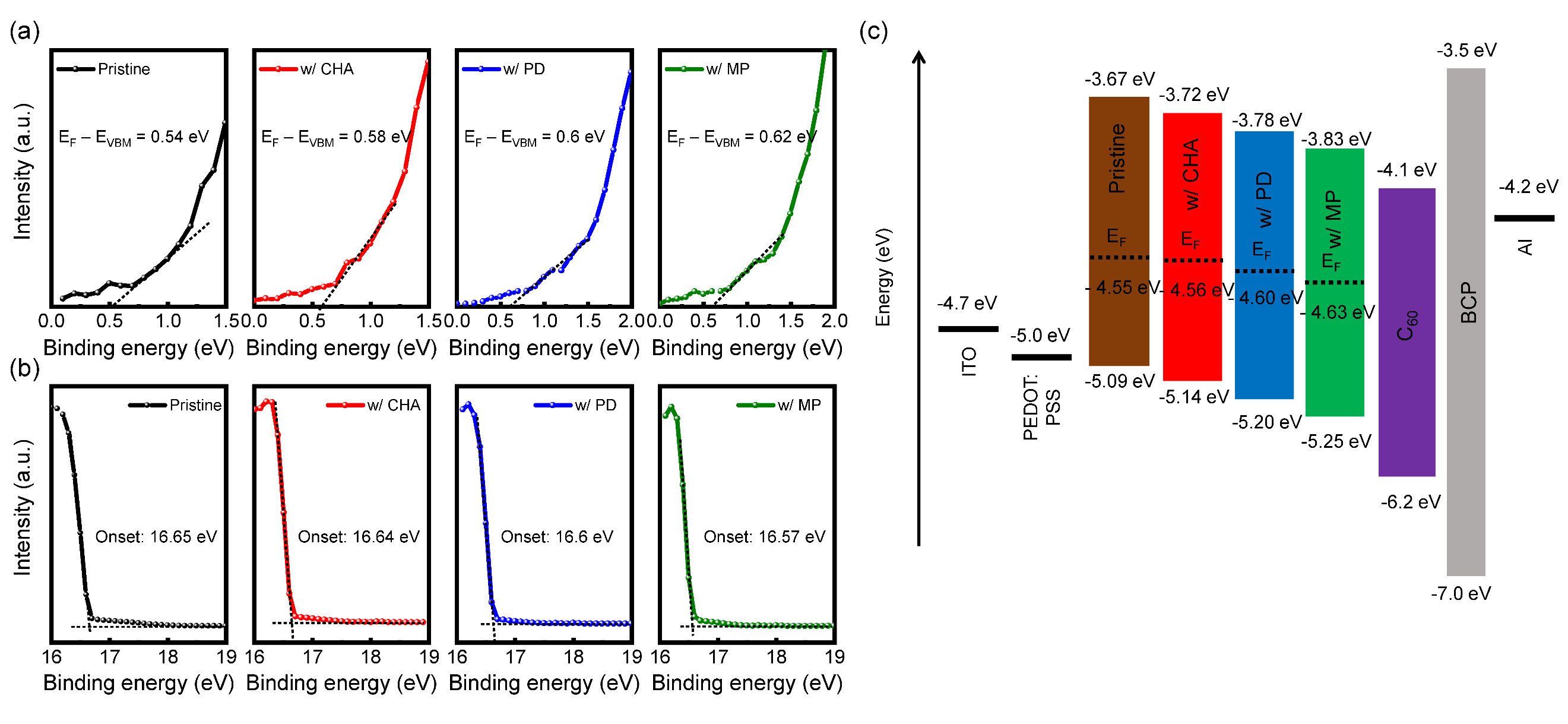


**Figure S15**. UPS spectra of (a) valence band and (b) secondary electron cutoff of the 3D and 2D/3D perovskite films. (c) Schematic diagram of the energy levels for corresponding PSCs.


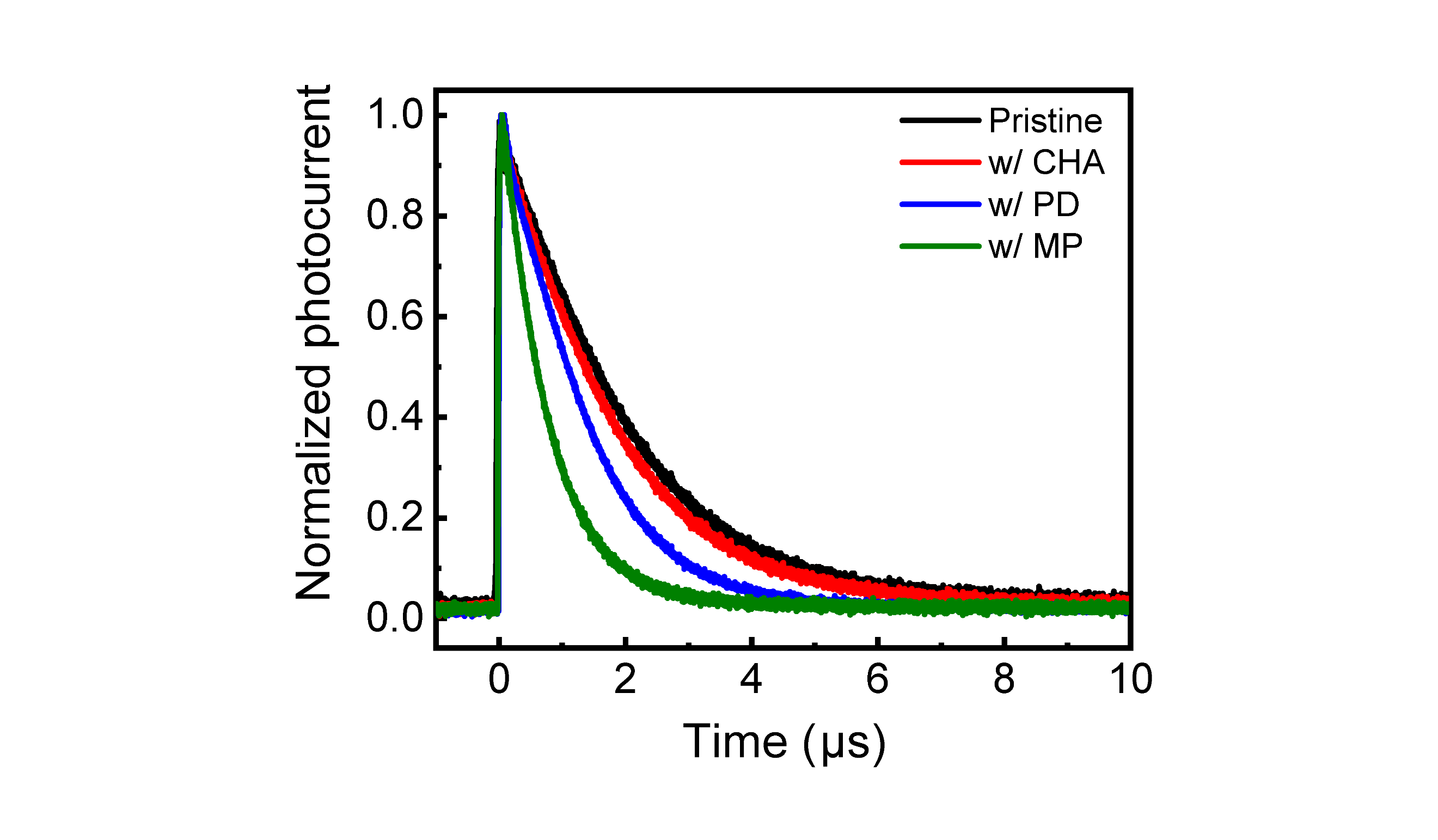


**Figure S16**. TPC measurements of the pristine and 2D/3D PSCs.


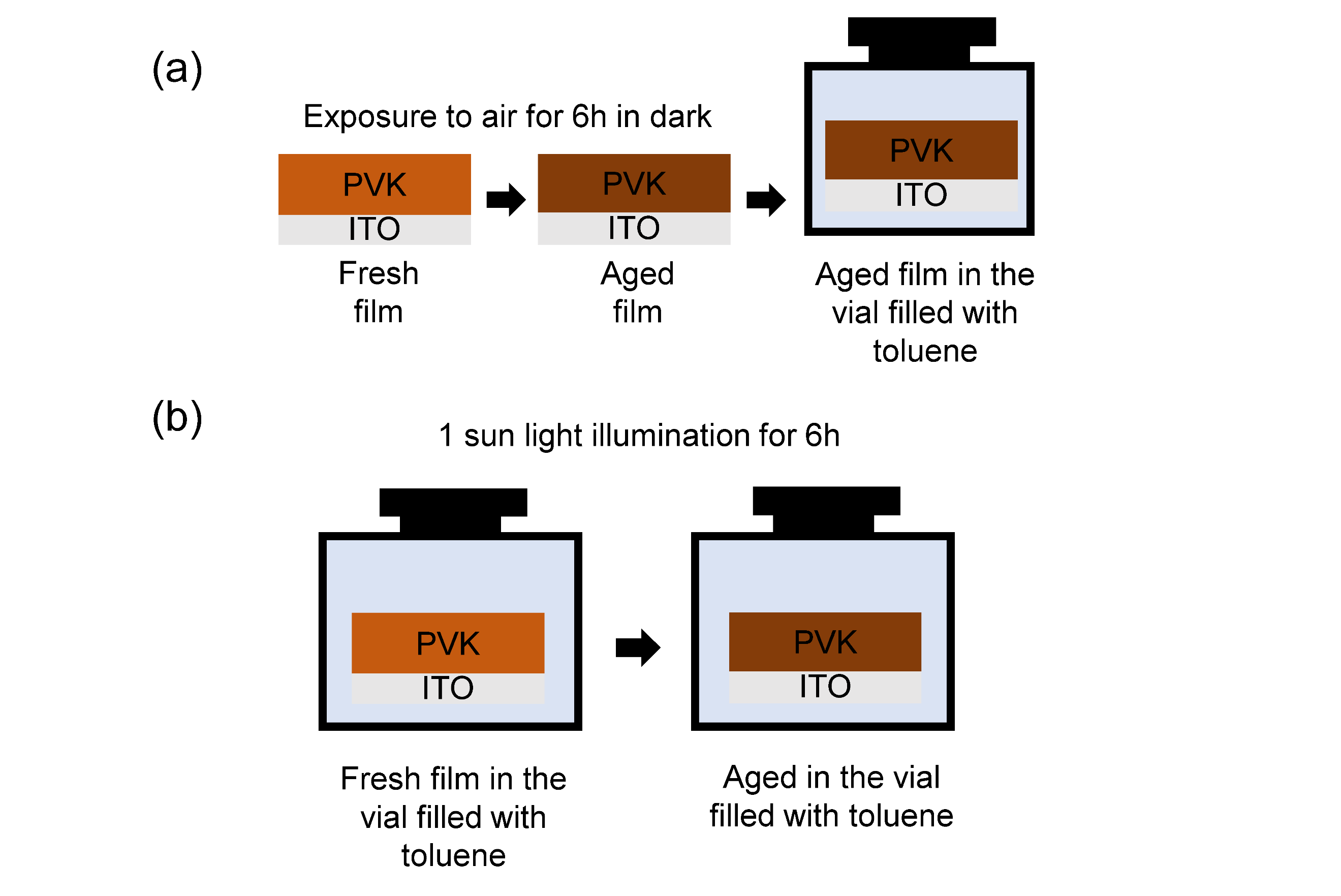


**Figure S17**. (a) Schematic illustration of the preparation of perovskite degradation products after exposure to air for 6h in dark, followed by dissolving in toluene to generate SnI_4_. (b) Schematic illustration of the preparation of perovskite degradation products dissolved in toluene after light illumination for 6h to generate I_2_. The whole procedure was done in a glovebox.


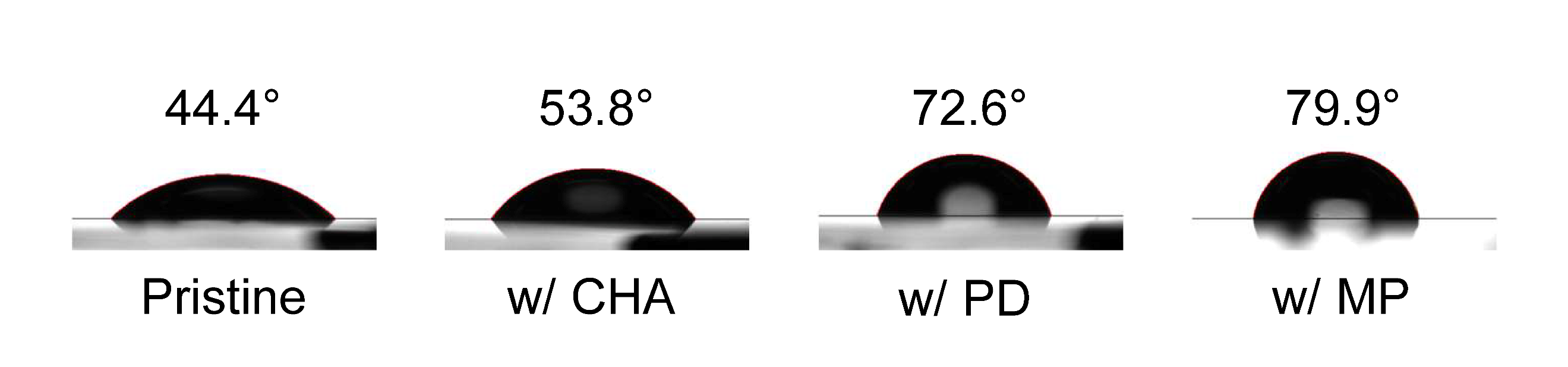


**Figure S18**. Water-contact angle measurements on top of the 3D and 2D/3D perovskite films.

**Table S1**. Calculated formation energy of neutral I and Sn vacancies in 3D and pure 2D perovskite.

| **Formation energy of defects (eV)** | **Types of perovskite** | | | |
| --- | --- | --- | --- | --- |
|  | **FASnI_3_** | **CHA_2_SnI_4_** | **PD_2_SnI_4_** | **MP_2_SnI_4_** |
| V_I_(e) | 1.436 | 2.285 | 2.624 | 2.683 |
| V_I_(a) | 1.436 | 3.245 | 3.321 | 3.364 |
| V_Sn_ | –1.344 | 1.835 | 1.944 | 2.482 |
